# Dynamic modelling of costs and health consequences of school closure during an influenza pandemic

**DOI:** 10.1186/1471-2458-12-962

**Published:** 2012-11-09

**Authors:** Yiting Xue, Ivar Sønbø Kristiansen, Birgitte Freiesleben de Blasio

**Affiliations:** 1 Department of Biostatistics, Institute of Basic Medical Sciences, University of Oslo, Oslo, Norway; 2 Department of Infectious Disease Epidemiology, Division of Infectious Disease Control, Norwegian Institute of Public Health, Oslo, Norway; 3 Department of Health Management and Health Economics, Institute of Health and Society, University of Oslo, Oslo, Norway

**Keywords:** Influenza pandemic, School closure, Costs, Benefits, Simulation

## Abstract

**Background:**

The purpose of this article is to evaluate the cost-effectiveness of school closure during a potential influenza pandemic and to examine the trade-off between costs and health benefits for school closure involving different target groups and different closure durations.

**Methods:**

We developed two models: a dynamic disease model capturing the spread of influenza and an economic model capturing the costs and benefits of school closure. Decisions were based on quality-adjusted life years gained using incremental cost-effectiveness ratios. The disease model is an age-structured SEIR compartmental model based on the population of Oslo. We studied the costs and benefits of school closure by varying the age targets (kindergarten, primary school, secondary school) and closure durations (1–10 weeks), given pandemics with basic reproductive number of 1.5, 2.0 or 2.5.

**Results:**

The cost-effectiveness of school closure varies depending on the target group, duration and whether indirect costs are considered. Using a case fatality rate (CFR) of 0.1-0.2% and with current cost-effectiveness threshold for Norway, closing secondary school is the only cost-effective strategy, when indirect costs are included. The most cost-effective strategies would be closing secondary schools for 8 weeks if *R*_*0*_=1.5, 6 weeks if *R*_*0*_=2.0, and 4 weeks if *R*_*0*_= 2.5. For severe pandemics with case fatality rates of 1-2%, similar to the Spanish flu, or when indirect costs are disregarded, the optimal strategy is closing kindergarten, primary and secondary school for extended periods of time. For a pandemic with 2009 H1N1 characteristics (mild severity and low transmissibility), closing schools would not be cost-effective, regardless of the age target of school children.

**Conclusions:**

School closure has moderate impact on the epidemic’s scope, but the resulting disruption to society imposes a potentially great cost in terms of lost productivity from parents’ work absenteeism.

## Background

Influenza pandemics occur at irregular intervals and cause significant mortality and morbidity as well as substantial economic losses [1]. School closure is a possible strategy for mitigating transmission during the early phase of a pandemic when vaccine is not yet available. School closure has three main consequences: reducing the total disease burden, postponing the peak of infection and lowering the peak prevalence of the disease. Postponing the pandemic increases the time available for strain-specific vaccine production and distribution, and allows for more time to prepare for the peak workload in health care settings. Lowering the peak of the pandemic reduces the risk for overloading of health services and shortage of health care personnel due to influenza sickness.

Schools are thought to play a special role in transmission due to high contact rates among school children combined with higher susceptibility among children compared with adults. During the A(H1N1) pandemic in 2009, the estimated infection rate among school children was significantly higher than that of the general population [2]. However, extended school closure is costly and may cause significant disruption to local communities by keeping working parents away from work and reducing school children’s learning time. Quantifying the costs and benefits of school closure might help inform pandemic policy making.

There is currently no consensus about the expected health benefits of school closure [3]. Previous studies have investigated the impact of school closure either by analysing data from previous pandemics and epidemics or by computer simulation. The historical data approach includes studies of the 1918 influenza pandemic and suggests that school closure, combined with other interventions, lowered the disease burden and that the timing and duration of such interventions mattered [4,5]. A 2009 study of eight European countries indicated that during holidays and weekends the social contact patterns of children and the basic reproductive number were reduced by almost a quarter [6]. However, little effect on transmission was observed during a two-week kindergarten and primary school closure in Hong Kong in 2008 [7]. The estimated impact of school closure from computer simulations varies widely depending on model assumptions about how children contribute to influenza transmission, virus transmissibility and illness threshold when school closure is triggered [8-12]. Only a limited number of studies have explored the cost of school closure. Two studies focused on productivity loss of care-taking parents suggest that school closure for 12 weeks may cost 0.2-1% of GDP in the UK [13], and 4 weeks closure 0.1-0.3% of GDP in the US [14]. To reduce the economic loss from working parents, reactive short-term (1–4 weeks) school closure has been studied, where schools are shut when ICU units reach peak demand [15], but the optimal timing of such interventions may be difficult. Some studies have combined cost estimates with micro-simulation models [16-19] or dynamic compartmental models [20]. While the assumptions used in the studies differ, the general picture in the cost-effectiveness is that school closure may be effective under high transmissibility, and/or high severity. Some of the studies were based on the characteristics of the 2009 H1N1 pandemic. Halder and co-workers [16] found that productivity losses due to sick leave and taking care of children when schools are closed were the dominating part of cost. A similar result was obtained in a study by Brown and co-workers [17] suggesting that the cost of school closure may far outweigh the cost saved from reducing the disease burden when the severity is low, regardless of the transmissibility.

In this study, we estimated potential costs and health benefits of school closure when implemented before substantial transmission of influenza among children has occurred (proactive school closure). We combined the cost estimates with a dynamic epidemiological transmission model, and determined the optimal closure strategy based on incremental cost-effectiveness ratios. Our study complements previous work on school closure by focusing on the age of the target school children, covering several scenarios for transmissibility, closure duration and severity. The study may be useful for public health authorities and may inform preparedness planning for future influenza pandemics.

## Methods

### Background

We modelled the impact of school closure in the context of a local community, using the capital city of Norway, Oslo, as the study setting. The city has a population size of 587 000, covering 12% of the Norwegian population. The unemployment rate is low (3.4%) and women’s participation in the labour force is high (70% of women aged 15–74 are employed) [21]. The education system is composed of primary school for children aged 6 to 12 years and secondary school for children aged 13 to 18 years. The attendance rate in kindergarten is approximately 90% for children aged 1 to 5 [21].

### The disease model

**Figure 1:**
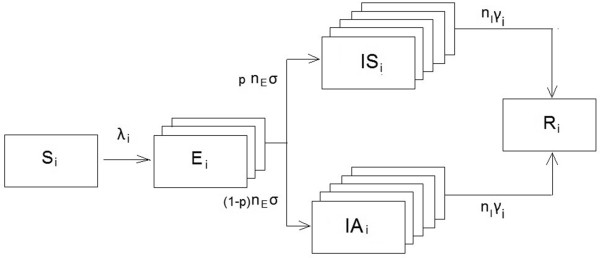
The dynamic influenza transmission model.

We considered a closed population of size *N*=587 000, ignoring demography (births, deaths and immigration) since influenza epidemics are of very short duration. We divided the population into six age groups (*i=1-6*): 1–5 years (6.7%), 6–12 years (7.2%), 13–18 years (6.9%), 19–39 years (36.6%), 40–64 years (30.5%) and 65+ years (12.2%). We modelled a pandemic influenza using a deterministic dynamic SEIR (*Susceptible-Exposed-Infected-Recovered*) model [22]. People in each age group are divided into four mutually exclusive compartments: susceptible, infected symptomatically, infected asymptomatically, and recovered with immunity/dead from influenza (Figure 1). People progress from one compartment to another at the rates determined by the contact pattern and characteristics of the virus.

A susceptible individual (*S*_*i*_) becomes infected according to the age-specific force of infection *λ*_*i*_. Newly infected individuals first enter the exposed state (*E*_*i*_) where they are infected, but not yet contagious, before developing either symptomatic infection (*IS*_*i*_) or asymptomatic infection (*IA*_*i*_). To obtain more realistic distributions of the exposed and infectious periods, we divided these periods into *n*_*i*_ stages, where the progression from each stage occurs at a rate *r*_*i*_ = *n*_*i*_/*D*_*i*_, where *D*_*i*_ is the mean duration of period *i* = *E*, *IS*, *IA*. This gives gamma distributed waiting times with shape parameters *k* = *n*_*i*_ and scale parameters *θ* = *D*_*i*_/*n*_*i*_. The mean duration of the exposed period was set to 1/*σ* = 1.9 days (17;18) and modelled in *n*_*E*_ = 3 stages. Individuals in the last exposed stage were assumed to be infectious with infectivity 50% compared to the infectivity of symptomatic infection, as viral shedding increases after one day following transmission [23]. We assumed that a proportion *p*=0.67 will become symptomatically infected while a proportion *(1-p)*=0.33 develop asymptomatic infection [24,25]. The average duration of the symptomatic infectious period was set to 1/*γ*_*c*_ =7 days for children (*i*=1, 2) and 1/*γ*_*a*_ = 5 days for adolescents/adults (*i*=3-6) [23,24,26] and modelled in *n*_*I*_ = 5 stages. Infectivity during the stages was set at 100%, 100%, 50%, 30% and 15% in accordance with data showing that viral transmission peaks during the early period after symptoms develop [23,27]. We assumed that asymptomatic infections are 50% as infectious per contact as symptomatic infections [23], but with similar duration and infectivity profile as symptomatic infections. However, other studies have found that asymptomatically infected individuals might be less important for transmission [28]. At the end of the infectious stage, people either recover or are removed from the system due to death. Individuals who have recovered from infection (*R*_*i*_) are assumed be protected from re-infection during the course of the simulation. The system can be described by a set of differential equations for each age group *i*=1-6:

$$\begin{array}{l} \frac{dS_{i}}{dt}=-S_{i}\lambda_{i}\\ \frac{dE_{1i}}{dt}=S_{i}\lambda_{i}-n_{E}\sigma E_{1i}\\ \frac{dE_{\mathrm{li}}}{dt}=n_{E}\sigma E_{\left(l-1\right)i}-n_{E}\sigma E_{\mathrm{li}}\;\forall l=2,3\\ \frac{dIA_{1i}}{dt}=(1-p)n_{E}\sigma E_{3i}-n_{I}\gamma_{i}IA_{1i}\;\\ \frac{dIA_{\mathrm{mi}}}{dt}=n_{I}\gamma_{i}IA_{\left(m-1\right)i}-n_{I}\gamma_{i}IA_{\mathrm{mi}}\;\forall m=\text{2…5}\;\\ \frac{dIS_{1i}}{dt}=pn_{E}\sigma E_{3i}-n_{I}\gamma_{i}IS_{1i}\;\\ \frac{dIS_{\mathrm{ni}}}{dt}=n_{I}\gamma_{i}IS_{\left(n-1\right)i}-n_{I}\gamma_{i}IS_{\mathrm{ni}}\;\forall n=\text{2…5}\;\\ \frac{dR_{i}}{dt}=n_{I}\gamma_{i}\left(IA_{5i}+IS_{5i}\right)\\ \lambda_{i}=\sum_{j=1}^{6}\beta_{\mathrm{ij}}\left(\alpha_{E}E_{j}+\sum_{k=1}^{5}\left(\alpha_{\mathrm{IA}}\left(k\right)IA_{\mathrm{kj}}+\alpha_{\mathrm{IS}}\left(k\right)IS_{\mathrm{kj}}\right)\right) \end{array}$$

Where *λ*_*i*_ is the per capita force of infection for a susceptible individual in age group *i* to become infected and *β*_*ij*_ is the transmission rate from age group *j* to age group *i* The age-specific force of infection *λ*_*i*_ is a product of age-specific contact rates, the prevalence of the infectious people (*I*_*i*_) and the probability of transmission given contact (*q*). We obtained the contact rates based on conversational data from a study in the Netherlands [29]. We employed a WAIFW matrix (“Who-acquires-infection-from-whom” matrix) based on the contact rates between age groups. The basic reproductive number (*R*_0_) was calculated as the largest eigenvalue in the next generation matrix (23). The basic reproductive number is “the average number of secondary cases arising from an average primary case in an entire susceptible population” [22]. Through varying the value of *q*, we can produce the desired *R*_*0*_.

The differential equations were solved numerically using a fourth-order Runge–Kutta method with adaptable step size in *Matlab* 2009. It is unclear whether cross-immunity from past exposure to influenza will provide protection against a future pandemic strain. We assumed that the population was fully susceptible to the novel pandemic strain at the beginning of the simulation. Transmission was initiated at day *t*_*i*_=1 by moving a proportion of 10^-6^ of susceptible in each age class into the exposed class. The simulation was run for a period of *t*=250 days.

The transmissibility of a future pandemic strain is a major source of uncertainty. For this reason, we tested the model with three different basic reproductive numbers *R*_*0*_=1.5, 2.0 and 2.5. The school closure intervention was initiated when the prevalence of symptomatic infections had reached 1% of the population and was assumed to have full impact from this point in time. In the baseline scenario (scenario A), we assumed a 90% reduction in contacts among isolated children/adolescents with individuals in their own age group and a 25% decrease in contacts with other age groups. We did not consider changes in the contact patterns of affected parents taking care of children at home in this baseline scenario.

### One-way sensitivity analysis

To account for some of the uncertainty in the model, we performed additional simulations varying assumptions about: the behaviours of care-taking parents, the behaviours of dismissed student during school closure and the case fatality rate (CFR).

In Scenario B, we introduced a 50% reduction in same age contacts among care-taking parents absent from work; in Scenario C we reduced the same age contact of dismissed children by 50% instead of 90% in the base case, and by 10% with other age groups instead of 25% to simulate low compliance among affected children; in Scenario D we increased the case fatality rate (CFR) by a factor of 10 compared to the baseline scenarios, using CFR of 1-2% in children and adults below 65 years similar to the level observed during the Spanish flu [30]; in Scenario E we reduced the CFR by a factor of 10 relative to the baseline scenarios, using CFR of 0.01-0.02% to simulate a mild pandemic. Finally, in Scenario F we modelled a pandemic with similar characteristics as the 2009 H1N1 pandemic. In these simulations, we assumed an *R*_*0*_ of 1.3. 60% of the populations in the 65+ year old age group and 10% of the 40–64 year old age group were assumed to have prior immunity. We also reduced the case fatality rate in accordance with Norwegian data showing that approximately 30 people died from H1N1 influenza (http://www.fhi.no/dokumenter/6cbae0eece.pdf).

### The economic model

The costs of school closure comprised parents’ productivity losses and students’ loss of learning. Avoided costs resulted from less use of health care resources, less loss of productivity and less use of energy in school buildings. Health benefits were expressed as gained quality-adjusted life-years (QALYs). Productivity loss due to illness and health benefits were included for cases of mortality and cases of morbidity. We used 2008 data (US$1.00=NOK7.00 [21]) for all economic calculations. All future costs and health outcomes were discounted by 4% as recommended by the Ministry of Health.

#### Costs of school closure

Absence from school means lost learning hours and potentially permanent loss of learning and income [31,32]. We searched the literature and databases, and contacted experts in education and educational economics. We were unable to identify any studies that directly address the issue of learning consequences of school closure. We assumed that this was the case only for students in upper secondary schools while children in kindergarten, primary and lower secondary school have no loss of learning from some weeks’ school closure. Most schools in Norway are public and free of charge, but some private schools offer upper secondary school education. Here, the tuition fee for one school year comprising 40 weeks was $8143, which is equivalent to $203 per week. We used this amount as an estimate of the value of lost learning.

School closure will keep working parents at home to care for children who are affected by the intervention. We assumed that students over 12 years do not need parental care during school closures. Similar to Sadique’s study [13], we assumed that only one parent is needed to care for children in a single household during school closure. Consequently, we distinguished between children living together with a single parent and with two parents. The percentages of both parents working were 66% among married couples with children and 78% among co-habitant couples with children (personal communication with *Statistics Norway*, 12 March, 2010). The percentage of working single parents was assumed to be the same as the percentage of working people in the same gender group (90% for men and 85% for women) [21]. We multiplied these percentages by the number of married couples, co-habitant couples and single parents, respectively. The sum of the products was taken as the number of individuals who would be absent from work during school closure.

We estimated the productivity losses from parents’ work absenteeism by multiplying the number of individuals that would need to be away from work during school closure with the number of days when schools are closed under different scenarios. The value of one day’s work was set equal to the national average wage rate (US$290 per day) plus 40%, which accounts for the value of productivity that is not returned to the worker as wages, including employer tax, payment for holiday and pension contributions.

#### Reduction of total cost due to school closure

The model outcome for symptomatically infected was divided into four types: mild cases who receive no medical care, moderate cases who receive outpatient service, severe cases who are hospitalized and fatal cases. Since the severity of a future pandemic is unknown, we used estimates of case fatality rates and health outcomes based on data from previous pandemics [33] (Table 1). We assumed that people with asymptomatic infection incur no economic costs, and therefore they were ignored in the economic analyses. The medical costs were estimated as the sum of mild, moderate and severe cases, multiplied by their respective unit costs. The unit costs were taken from a recent study of influenza costs in Norway [34].

**Table 1:** Model parameters

	**Mean**	**Distribution**	**Parameter**	**References**
**Demographic data**				
Population by age				^15^
1--5 years old	6.63%			
6—12 years old	7.17%			
13—19 years old	6.86%			
20—39 years old	36.65%			
40—64 years old	30.46%			
65+ years old	12.24%			
Percentage of adult population affected by school closure:				^15^
kindergarten (1-5 years old)	4.54%			
kindergarten/primary school (1-11 years old)	10%			
**Disease parameters**				
Basic reproductive number (*R*_*0*_)	1.5, 2.0, 2.5			^31; 32; 8^
Mean duration of exposed period	1.9 days			^17; 18^
Mean duration of infectious period	7 days (<12 years) 5 days (12+ years)			^17; 18; 19^
Proportion asymptomatic (*p*)	33%			
Infectivity (last exposed stage)	50%			^19^
Infectivity (in the five infectious stages)	100%, 100%, 50%, 30%, 15%			^19;20^
**Mixing assumptions**				
**Scenario A (baseline)**				
Reduction in contact rate between dismissed children of same/other age groups	90%/25%			
Reduction in contact rate among care-taking parents and same age group	0%			
**Scenario B**				
Reduction in contact rate between dismissed children of same/other age groups	90%/25%			
Reduction in contact rate among care-taking parents and same age group	50%			
**Scenario C**				
Reduction in contact rate between dismissed children of same/other age groups	50%/10%			
Reduction in contact rate among care-taking parents and same age group	0%			
**Disease outcomes**				
Outcomes per 1000 cases by age groups^a^				^25^
Outpatient	(534, 389, 497)	Uniform	((494-574), (369-410), (487-506))	
Inpatient	(4, 8, 29)		((1-8), (2-13), (21-37))	
Death	(1, 2, 13)		((0-2),(0-4),(11-15))	
**Economic parameters**				
Cost of energy saving (1000 US$)	1 439	Gamma^b^	α=16; β=90	*Oslo Municipality*
Cost of lost learning (1000 US$)	25 797	Gamma	α=16; β=1 612	*Bjørknes private school*
Proportion of productivity loss catching up	15%	Uniform	range [0: 30%]	
Average cost per self-care person (US$)	43	Normal	σ=3.57	^26^
Average cost per out-patient (US$)	59	Normal	σ=4.92	*Den norske legeforening*
Average cost per in-patient (US$)	5 211	Normal	σ=434	^26^
Average wage per day (US$)	290	Normal	σ=24	^15^

^a^Age groups were grouped by 1—18 years old, 19—64 years old and 65+years old.

^b^$f\left(x;k,\theta \right)=x^{k-1}\frac{e^{-x/\theta }}{\theta^{k}\Gamma \left(k\right)}$ where *Γ* is the Gamma function.

Loss of productivity associated with influenza has two components: the loss of working hours for the symptomatically infected and the loss of potential productivity for the fatal cases. Productivity losses due to morbidity were valued in the same way as parents’ work absenteeism. Productivity losses due to mortality were valued according to the remaining life expectancy at the relevant ages, discounted by 4% and with the assumption that people participate in the work force until age 65.

The avoided school heating cost was estimated using data from the *Educational Buildings and Property Department* in Oslo municipality.

#### Health benefits

Assuming that school closure will reduce the number of symptomatic and fatal influenza cases, we expressed the health benefits from school closure in terms of quality-adjusted life years (QALYs). For those who are symptomatically infected, we used utility scores from a Canadian study [35]. These utility scores represent the utility people have on each of the seven days since the onset (0 for worst possible health and 1 for normal health). The utilities are 0.41, 0.47, 0.58, 0.67, 0.73, 0.78 and 0.81 for day 1 to day 7, respectively. For those who died due to the illness, the QALY loss was calculated from the remaining life expectancy at the age of death predicted by the disease model and the discount factor.

#### Intervention strategy scenarios

We explored the costs and benefits of intervention policies with different durations (from 1 to 10 weeks) and for different target groups (closing kindergarten alone, primary school alone, secondary school alone, kindergarten and primary school or all three).

### Uncertainty in cost-effectiveness estimates

To quantify the uncertainty in the cost-effectiveness ratios, we performed a probabilistic sensitivity analysis (number of simulations=1000) on the selected strategy for *R*_*0*_= 1.5, 2.0 and 2.5, incorporating the uncertainty in the demographic parameters, disease parameters, disease outcomes and economic parameters (Table 1). In addition, we reduced the work loss of care-taking parents by 0-30% (uniform distribution) assuming that some children were cared for by relatives or other persons, or that part of their work loss could be carried out through work from home or through work at a later time. The results were presented graphically by means of cost-effectiveness acceptability curves (Additional file 1: e-Figure 1).

## Results

### Epidemiological impact of school closure

**Figure 2:**
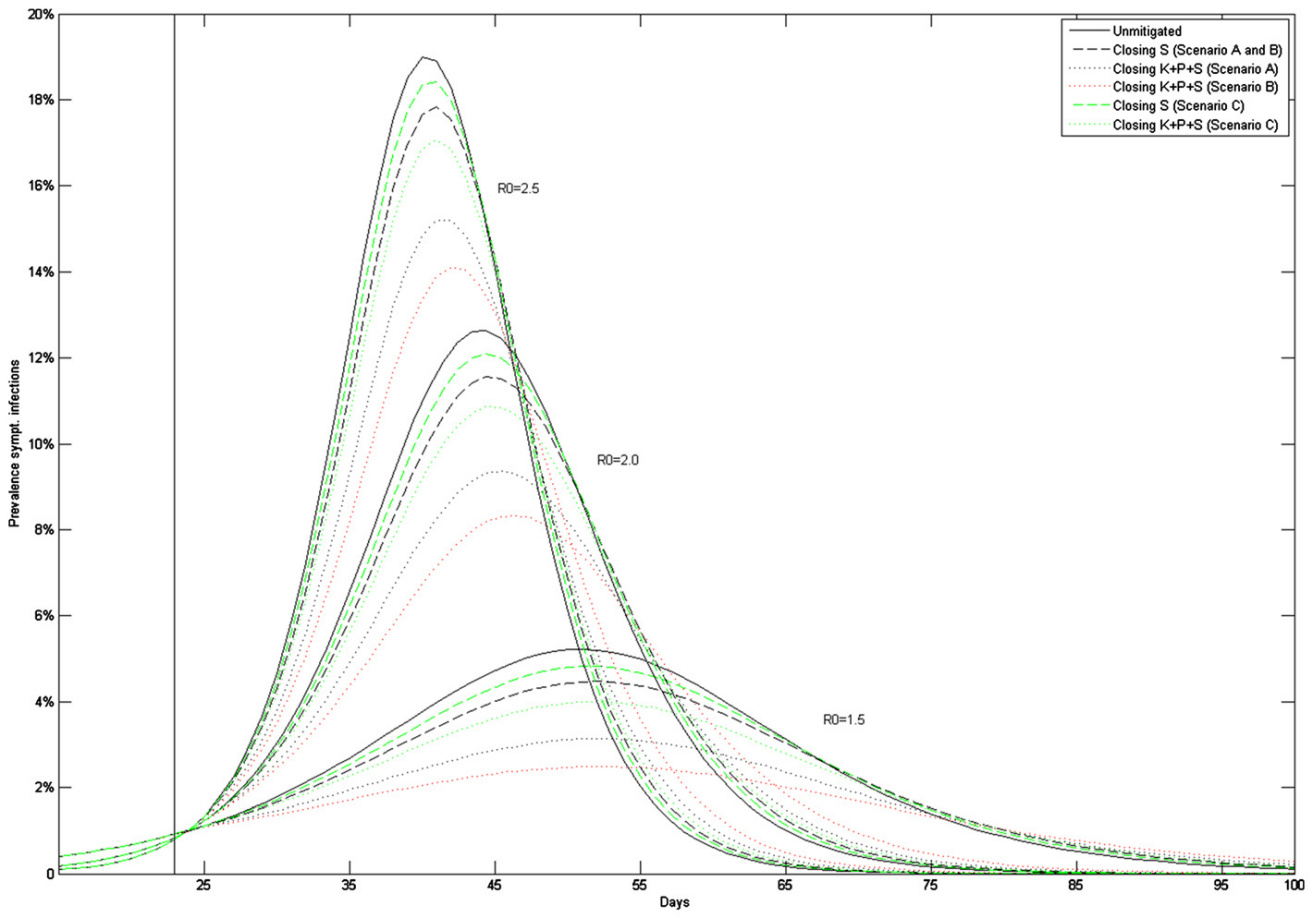
**Epidemic curves showing the prevalence of symptomatic infections for unmitigated pandemic versus implementing a 12-week school closure with *R***_***0***_**=1.5, 2.0 and 2.5.**

**Figure 3:**
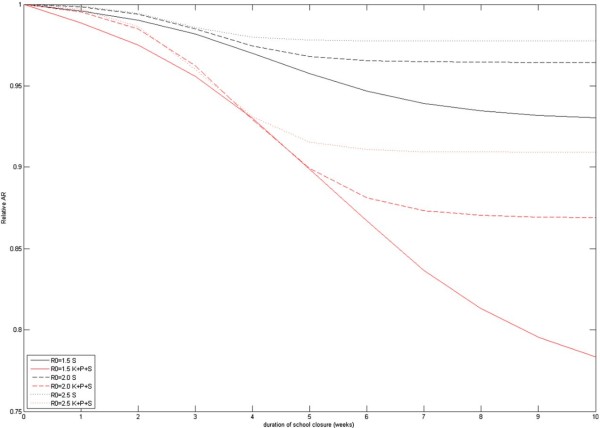
The relative attack rate compared to an unmitigated pandemic as function of school closure duration (number of closure weeks).

Figures 2, 3 show the epidemiological results of school closure. In the absence of intervention, our baseline model predicts 216 000, 300 000 and 340 000 symptomatic infections in the Oslo population for *R*_*0*_ =1.5, 2.0 and 2.5 pandemics, corresponding to clinical attack rates (AR) of 37%, 51% or 58%, respectively (Table 2). The relative effectiveness of the interventions increased with lower *R*_*0*_ values but required longer closure time to achieve the health benefits (Figure 3). School closure lowers the attack rate with up to 7-22%, 4-13% and 2-9% with *R*_*0*_=1.5, 2.0 or 2.5; these reductions are achieved after approximately 10, 8 and 7 weeks of closure (Figure 3). The peak prevalence of symptomatic infections was reduced correspondingly with up to 7-36%, 6-26% and 5-20%. To reach maximum reduction, school closure must be maintained for some weeks and beyond the point in time when the mitigated pandemic passes through its natural peak (Additional file 1: e-Figure 2). If schools are re-opened earlier, the pandemic will rebound. This will also happen if the intervention stops in the wake of the pandemic, provided the effective reproductive number of the *un-mitigated* pandemic is still above 1. Consequently, the maximum delay of the peak occurred for intermediate closure durations. The peak was delayed by up to 8–10 days (*R*_*0*_ =1.5), and to 4–5 days for *R*_*0*_ =2.0, 2.5. To avoid restarting the epidemic, we found that closure must be effective for at least 3–4 week for *R*_*0*_ =1.5, and 2–3 weeks when the transmissibility is higher.

**Table 2:** **Disease outcomes given *R***_***0***_**=1.5, 2.0 and 2.5**

**School closure of 12 weeks**	**R0=1.5**				**R0=2.0**				**R0=2.5**			
	**outp.**	**inp.**	**deaths**	**AR(%)**	**outp.**	**inp.**	**deaths**	**AR(%)**	**outp.**	**inp.**	**deaths**	**AR(%)**
**No intervention**	92779	1929	584	37	128932	2738	844	51	146088	3150	983	58
Scenario A (baseline)												
K	87388	1846	560	35	123904	2673	825	49	141642	3098	968	56
P	83081	1779	540	33	121245	2638	815	49	140075	3080	962	56
S	85718	1813	550	34	123784	2665	822	49	142328	3101	968	57
K+P	77605	1692	514	31	115823	2566	793	47	135161	3022	945	54
K+P+S	69989	1559	474	29	109800	2477	767	44	130661	2962	927	53
	**SENSITIVITY ANALYSIS**											
	**R0=1.5**				**R0=2.0**				**R0=2.5**			
	**outp.**	**inp.**	**deaths**	**AR(%)**	**outp.**	**inp.**	**deaths**	**AR(%)**	**outp.**	**inp.**	**deaths**	**AR(%)**
Scenario B												
K	85200	1798	546	34	122669	2645	817	49	140911	3082	963	56
P	79765	1707	519	32	119377	2597	803	48	138986	3056	955	55
S	85718	1813	550	34	123784	2665	822	49	142328	3101	968	57
K+P	71608	1559	475	29	112224	2487	770	45	133028	2975	932	53
K+P+S	64030	1423	434	26	105671	2387	740	43	128221	2910	912	52
Scenario C												
K	89954	1885	572	36	126502	2707	835	50	144110	3127	976	57
P	87441	1847	560	35	125354	2691	830	50	143677	3121	974	57
S	89346	1873	568	36	126696	2706	835	50	144607	3131	977	57
K+P	84498	1801	547	34	122774	2657	820	49	141574	3097	967	56
K+P+S	80744	1738	528	33	120292	2621	810	48	139934	3075	961	56

Outp= outpatient. Inp= inpatient. AR=attack rate.

Scenario A is the base case scenario; scenario B included a 50% reduction in contacts among care-taking parents absent from work based on scenario A; scenario C reduced the compliance to 50% from scenario A.

The baseline scenarios gave an estimated 93 000–147 000 outpatient visits, 1 900–3 100 hospitalizations and 590–990 deaths (Table 2). The simulation runs showed that a 12-week school closure would reduce the attack rate by up to 22%, 14% and 7% for *R*_*0*_=1.5, 2.0 and 2.5 pandemics. The reductions in disease outcomes followed the reductions in attack rate, with slightly higher reductions in outpatients (6%–25%) and slightly lower reductions in inpatients and deaths (4%–20%) for a 12-week closure with *R*_*0*_=1.5, 2.0 or 2.5 in the base case.

### Economic impact

Without school closure, the total health care costs would be $21 million, $29 million and $33 million, productivity losses due to mortality would be $313 million, $428 million and $480 million and productivity losses due to morbidity $102 million, $139 million and $155 million, for basic reproductive numbers of 1.5, 2.0 and 2.5 (Tables 3, 4 and 5). Depending on the type and duration of school closure, the cost of lost learning would be $0–32 million, while the cost of lost productivity were in the range of $0–630 million, and reduction in school heating costs varied between $0.18 and 5.4 million. The total influenza related costs would range from $435 million to $1285 million from the societal perspective (Tables 3, 4 and 5).

**Table 3:** **Cost and health outcome according to type and duration of school closure when *R***_***0***_**=1.5**

**Target school**	**Duration (weeks)**	**Cost of lost learning ($1000)**	**Lost productivity due to school closure ($1000)**	**Energy savings ($1000)**	**Health care costs ($1000)**	**Lost productivity due to fatal cases ($1000)**	**Lost productivity due to sickness ($1000)**	**Total cost ($1000)**	**QALY gains (compared to no intervention)**	**Cost per QALY (compared to no intervention)**	**ICER**
0	0	0	0	0	20 591	312 958	101 576	435 125	0		
3	6	19 350	0	1 080	19 557	298 239	97 846	433 912	507	−2 395	
3	7	22 575	0	1 260	19 410	296 139	97 312	434 175	579	−1 641	3 648
3	5	16 125	0	900	19 766	301 213	98 600	434 804	404	−796	Dominated
**3**	**8**	**25 800**	**0**	**1 440**	**19 318**	**294 825**	**96 978**	**435 481**	**624**	**570**	**28 929**
3	4	12 900	0	720	20 008	304 661	99 474	436 323	286	4 193	Dominated
3	1	3 225	0	180	20 509	311 792	101 278	436 625	40	37 316	Dominated
3	9	29 025	0	1 620	19 264	294 064	96 784	437 517	650	3 679	77 819
3	3	9 675	0	540	20 235	307 897	100 293	437 560	174	13 962	Dominated
3	2	6 450	0	360	20 403	310 287	100 897	437 678	92	27 727	Dominated
3	10	32 250	0	1 800	19 237	293 672	96 684	440 043	664	7 412	187 991
2	1	0	26 795	188	20 495	311 614	101 261	459 977	47	531 474	
1	1	0	36 194	174	20 530	312 120	101 383	470 054	29	1 193 056	
2	2	0	53 591	376	20 385	310 094	100 909	484 603	100	496 745	
4	1	0	62 989	362	20 440	310 857	101 085	495 009	73	817 647	
5	1	3 225	62 989	542	20 367	309 817	100 816	496 672	109	564 499	
1	2	0	72 388	348	20 453	311 044	101 138	504 674	67	1 039 847	
2	3	0	80 386	564	20 210	307 651	100 342	508 024	185	395 026	
2	4	0	107 181	752	19 950	304 039	99 504	529 922	310	305 814	
1	3	0	108 582	522	20 323	309 257	100 729	538 369	129	798 460	
2	5	0	133 976	940	19 645	299 793	98 516	550 991	457	253 333	
4	2	0	125 979	724	20 263	308 401	100 517	554 436	159	751 538	
5	2	6 450	125 979	1 084	20 109	306 200	99 944	557 598	234	522 386	
1	4	0	144 776	696	20 148	306 836	100 173	571 237	214	636 557	
2	6	0	160 772	1 128	19 356	295 758	97 575	572 332	597	229 715	
2	7	0	187 567	1 316	19 117	292 419	96 793	594 579	713	223 637	
1	5	0	180 970	870	19 971	304 374	99 606	604 051	300	563 630	
4	3	0	188 968	1 086	19 989	304 590	99 632	612 092	291	607 281	
5	3	9 675	188 968	1 626	19 736	300 952	98 673	616 377	416	435 391	
2	8	0	214 362	1 504	18 961	290 230	96 279	618 327	789	232 250	
1	6	0	217 164	1 044	19 812	302 170	99 095	637 197	377	536 585	
2	9	0	241 157	1 692	18 858	288 799	95 942	643 065	838	248 040	
4	4	0	251 957	1 448	19 608	299 290	98 394	667 801	476	489 135	
2	10	0	267 953	1 880	18 803	288 023	95 759	668 657	865	269 909	
1	7	0	253 358	1 218	19 703	300 654	98 742	671 239	429	549 816	
5	4	12 900	251 957	2 168	19 239	293 960	96 967	672 854	658	361 133	
1	8	0	289 552	1 392	19 639	299 767	98 536	706 101	460	588 611	
4	5	0	314 946	1 810	19 151	292 916	96 892	722 096	697	411 700	
5	5	16 125	314 946	2 710	18 630	285 363	94 847	727 200	955	305 707	
1	9	0	325 746	1 566	19 605	299 302	98 427	741 514	477	642 905	
4	6	0	377 936	2 172	18 702	286 631	95 396	776 493	915	373 065	
1	10	0	361 940	1 740	19 585	299 025	98 363	777 173	486	703 475	
5	6	19 350	377 936	3 252	18 018	276 690	92 681	781 422	1 255	275 997	
4	7	0	440 925	2 534	18 330	281 412	94 143	832 277	1 096	362 429	
5	7	22 575	440 925	3 794	17 426	268 283	90 555	835 970	1 544	259 543	
4	8	0	503 914	2 896	18 058	277 580	93 217	889 873	1 228	370 185	
5	8	25 800	503 914	4 336	16 965	261 700	88 870	892 913	1 771	258 490	
4	9	0	566 903	3 258	17 885	275 152	92 627	949 309	1 312	391 782	
5	9	29 025	566 903	4 878	16 627	256 878	87 624	952 179	1 937	266 957	
4	10	0	629 893	3 620	17 789	273 802	92 297	1 010 161	1 359	423 098	
5	10	32 250	629 893	5 420	16 386	253 424	86 726	1 013 259	2 056	281 259	

Note: The maximum willingness to pay is set to be NOK 500,000 or US$71,500 based on the government guidance^28^. The most cost-effective option is shown with bold font.

**Table 4:** **Cost and health outcome according to type and duration of school closure when *R***_***0***_**=2.0**

**Target school**	**Duration (weeks)**	**Cost of lost learning ($1000)**	**Lost productivity due to school closure ($1000)**	**Energy savings ($1000)**	**Health care costs ($1000)**	**Lost productivity due to fatal cases ($1000)**	**Lost productivity due to sickness ($1000)**	**Total cost ($1000)**	**QALY gains (compared to no intervention)**	**Cost per QALY (compared to no intervention)**	**ICER**
0	0	0	0	0	28 890	428 137	138 654	595 682			
3	4	12 900	0	720	28 215	419 135	136 843	596 374	321	2 155	
3	5	16 125	0	900	28 049	416 920	136 411	596 604	400	2 306	2 921
3	1	3 225	0	180	28 846	427 542	138 529	597 961	21	106 854	
3	3	9 675	0	540	28 491	422 813	137 570	598 009	190	12 224	Dominated
**3**	**6**	**19 350**	**0**	**1 080**	**27 985**	**416 062**	**136 245**	**598 562**	**431**	**6 686**	**64 224**
3	2	6 450	0	360	28 732	426 018	138 216	599 056	76	44 470	Dominated
3	7	22 575	0	1 260	27 964	415 780	136 190	601 248	441	12 628	267 404
3	8	25 800	0	1 440	27 957	415 695	136 173	604 186	444	19 161	975 711
3	9	29 025	0	1 620	27 955	415 672	136 169	607 201	445	25 907	3 654 485
3	10	32 250	0	1 800	27 955	415 664	136 167	610 236	445	32 714	11 358 909
2	1	0	26 795	188	28 844	427 532	138 545	621 528	22	1 179 444	
1	1	0	36 194	174	28 853	427 657	138 575	631 105	17	2 029 542	
2	2	0	53 591	376	28 735	426 112	138 297	646 358	73	691 761	
4	1	0	62 989	362	28 810	427 096	138 472	657 005	38	1 622 566	
5	1	3 225	62 989	542	28 769	426 546	138 354	659 342	58	1 107 041	
1	2	0	72 388	348	28 752	426 362	138 363	665 517	65	1 082 646	
2	3	0	80 386	564	28 481	422 818	137 732	668 853	192	380 871	
2	4	0	107 181	752	28 108	417 981	136 924	689 442	366	256 079	
1	3	0	108 582	522	28 535	423 576	137 909	698 080	166	618 250	
2	5	0	133 976	940	27 795	413 930	136 260	711 021	512	225 466	
4	2	0	125 979	724	28 618	424 608	138 047	716 527	128	944 416	
5	2	6 450	125 979	1 084	28 494	422 945	137 687	720 470	187	665 879	
1	4	0	144 776	696	28 275	420 231	137 363	729 949	287	467 968	
2	6	0	160 772	1 128	27 636	411 869	135 925	735 074	585	238 117	
2	7	0	187 567	1 316	27 576	411 079	135 797	760 703	614	268 896	
1	5	0	180 970	870	28 108	418 079	137 011	763 298	365	459 401	
4	3	0	188 968	1 086	28 179	418 930	137 085	772 075	333	529 302	
5	3	9 675	188 968	1 626	27 906	415 233	136 270	776 425	465	388 791	
2	8	0	214 362	1 504	27 557	410 834	135 758	787 007	622	307 365	
1	6	0	217 164	1 044	28 044	417 253	136 876	798 292	395	513 250	
2	9	0	241 157	1 692	27 551	410 765	135 746	813 528	625	348 587	
4	4	0	251 957	1 448	27 555	410 851	135 722	824 637	624	366 634	
5	4	12 900	251 957	2 168	27 050	403 997	134 214	827 950	868	267 629	
1	7	0	253 358	1 218	28 022	416 976	136 830	833 968	405	588 668	
2	10	0	267 953	1 880	27 550	410 744	135 743	840 109	626	390 639	
1	8	0	289 552	1 392	28 017	416 902	136 818	869 897	407	672 986	
4	5	0	314 946	1 810	27 034	404 084	134 582	878 837	868	326 324	
5	5	16 125	314 946	2 710	26 272	393 758	132 340	880 731	1 234	230 988	
1	9	0	325 746	1 566	28 015	416 881	136 815	905 892	408	759 953	
4	6	0	377 936	2 172	26 753	400 417	133 965	936 898	999	341 470	
5	6	19 350	377 936	3 252	25 798	387 502	131 192	938 526	1 457	235 261	
1	10	0	361 940	1 740	28 015	416 876	136 814	941 904	408	847 747	
4	7	0	440 925	2 534	26 645	399 011	133 728	997 774	1 050	383 078	
5	7	22 575	440 925	3 794	25 597	384 843	130 703	1 000 849	1 552	261 045	
4	8	0	503 914	2 896	26 611	398 573	133 654	1 059 855	1 065	435 700	
5	8	25 800	503 914	4 336	25 521	383 838	130 518	1 065 255	1 588	295 719	
4	9	0	566 903	3 258	26 600	398 435	133 631	1 122 311	1 070	492 048	
5	9	29 025	566 903	4 878	25 494	383 489	130 454	1 130 488	1 600	334 181	
4	10	0	629 893	3 620	26 597	398 391	133 623	1 184 883	1 072	549 693	
5	10	32 250	629 893	5 420	25 486	383 386	130 435	1 196 030	1 604	374 276	

**Table 5:** **Cost and health outcome according to type and duration of school closure when *R***_***0***_**=2.5**

**Target school**	**Duration (weeks)**	**Cost of lost learning ($1000)**	**Lost productivitydue to school closure ($1000)**	**Energy savings ($1000)**	**Health care costs ($1000)**	**Lost productivity due to fatal cases ($1000)**	**Lost productivity due to sickness ($1000)**	**Total cost ($1000)**	**QALY gains (compared to no intervention)**	**Cost per QALY (compared to no intervention)**	**ICER**
0	0	0	0	0	32 961	479 607	155 079	667 646			
3	1	3 225	0	180	32 928	479 185	155 005	670 162	16	160 991	
3	3	9 675	0	540	32 544	474 295	154 205	670 179	195	12 994	
**3**	**4**	**12 900**	**0**	**720**	**32 367**	**472 045**	**153 864**	**670 456**	**277**	**10 150**	**3 380**
3	2	6 450	0	360	32 801	477 565	154 728	671 184	75	47 003	Dominated
3	5	16 125	0	900	32 318	471 424	153 771	672 739	299	17 011	101 226
3	6	19 350	0	1 080	32 308	471 296	153 752	675 626	304	26 248	620 315
3	7	22 575	0	1 260	32 306	471 271	153 749	678 641	305	36 059	3 386 921
3	8	25 800	0	1 440	32 306	471 267	153 748	681 681	305	46 005	20 007 697
3	9	29 025	0	1 620	32 306	471 266	153 748	684 725	305	55 979	126 703 892
3	10	32 250	0	1 800	32 306	471 266	153 748	687 770	305	65 955	289 245 859
2	1	0	26 795	188	32 929	479 210	155 022	693 768	15	1 764 356	
1	1	0	36 194	174	32 929	479 216	155 031	703 195	15	2 442 399	
2	2	0	53 591	376	32 811	477 750	154 823	718 598	69	738 185	
4	1	0	62 989	362	32 899	478 844	154 977	729 347	28	2 168 767	
5	1	3 225	62 989	542	32 871	478 484	154 908	731 936	42	1 538 432	
1	2	0	72 388	348	32 797	477 600	154 839	737 276	75	934 187	
2	3	0	80 386	564	32 504	473 932	154 337	740 595	210	348 001	
2	4	0	107 181	752	32 174	469 835	153 856	762 295	359	263 354	
1	3	0	108 582	522	32 532	474 332	154 456	769 379	196	520 297	
2	5	0	133 976	940	32 013	467 836	153 631	786 516	432	275 032	
4	2	0	125 979	724	32 671	476 022	154 614	788 561	133	907 803	
5	2	6 450	125 979	1 084	32 558	474 572	154 337	792 811	187	670 647	
1	4	0	144 776	696	32 315	471 663	154 148	802 206	294	457 624	
2	6	0	160 772	1 128	31 970	467 302	153 571	812 487	452	320 722	
1	5	0	180 970	870	32 242	470 771	154 046	837 160	327	518 523	
2	7	0	187 567	1 316	31 962	467 200	153 560	838 973	455	376 283	
4	3	0	188 968	1 086	32 117	469 165	153 760	842 924	386	453 852	
5	3	9 675	188 968	1 626	31 806	465 143	153 021	846 987	533	336 228	
2	8	0	214 362	1 504	31 960	467 181	153 558	865 557	456	434 015	
1	6	0	217 164	1 044	32 226	470 573	154 023	872 943	334	614 247	
2	9	0	241 157	1 692	31 960	467 177	153 557	892 159	456	492 180	
4	4	0	251 957	1 448	31 531	461 895	152 891	896 827	653	351 186	
5	4	12 900	251 957	2 168	30 971	454 663	151 625	899 947	916	253 572	
1	7	0	253 358	1 218	32 224	470 539	154 019	908 922	335	719 170	
2	10	0	267 953	1 880	31 960	467 176	153 557	918 766	456	550 486	
1	8	0	289 552	1 392	32 223	470 532	154 019	944 934	336	825 928	
4	5	0	314 946	1 810	31 242	458 297	152 470	955 145	784	366 760	
5	5	16 125	314 946	2 710	30 530	449 119	150 906	958 916	1 118	260 600	
1	9	0	325 746	1 566	32 223	470 531	154 019	980 953	336	933 116	
4	6	0	377 936	2 172	31 161	457 290	152 353	1 016 567	821	425 217	
1	10	0	361 940	1 740	32 223	470 531	154 019	1 016 972	336	1 040 374	
5	6	19 350	377 936	3 252	30 395	447 425	150 689	1 022 542	1 179	300 960	
4	7	0	440 925	2 534	31 145	457 092	152 330	1 078 958	828	496 882	
5	7	22 575	440 925	3 794	30 360	446 990	150 633	1 087 690	1 195	351 506	
4	8	0	503 914	2 896	31 142	457 055	152 326	1 141 540	829	571 548	
5	8	25 800	503 914	4 336	30 354	446 910	150 623	1 153 266	1 198	405 402	
4	9	0	566 903	3 258	31 142	457 047	152 325	1 204 158	829	646 844	
5	9	29 025	566 903	4 878	30 353	446 893	150 621	1 218 917	1 199	459 966	
4	10	0	629 893	3 620	31 141	457 045	152 324	1 266 783	829	722 298	
5	10	32 250	629 893	5 420	30 352	446 889	150 620	1 284 584	1 199	514 689	

Health benefits from school closure would range from 15 QALYs to 2056 QALYs depending on *R*_*0,*_ the age target group and the duration of school closure (Tables 3, 4 and 5). Our results indicate that in the baseline scenario, closing secondary schools for 8, 6 and 4 weeks, when *R*_*0*_ is 1.5, 2.0 and 2.5 respectively, is the most cost-effective strategy when indirect costs are accounted for. Closing secondary schools is cost-effective given a wide range of cost-effective threshold ratios, as shown by cost-effectiveness acceptability curves (Additional file 1: e-Figure 1). The strategy of closing secondary was also cost-effective for varying closure durations (data not shown).

### Sensitivity analyses

The sensitivity analyses confirm that closing secondary schools is the optimal strategy from a societal perspective, unless the case fatality rate (CFR) is very high.

**Scenario B:** Reduced (adult-adult) contact among care-taking parents. We found increased effect of school closure relative to the baseline scenarios. The estimated reduction in the attack rate compared to an unmitigated pandemic was 8-30%, 4-16%, and 3-10%, for *R*_*0*_=1.5, 2.0 and 2.5 pandemics, respectively (Table 2). The corresponding optimal strategies were closing secondary schools with durations of 8 weeks, 6 weeks and 4 weeks, identical to the findings in the baseline scenario (Additional file 1: e-Table 1; I-III).

**Scenario C:** Reduced compliance of dismissed children/students to stay at home. The simulations showed an overall small effect of school closure. The estimated maximum reduction in the attack rate compared to an unmitigated pandemic ranged between 3-11%, 2-6% and 2-3% for *R*_*0*_=1.5, 2.0 and 2.5, respectively (Table 2). The optimal strategies were closing secondary schools for 7, 4, and 3 weeks (Additional file 1: e-Table 2; I-III), indicating a shorter optimal period of one week compared with the baseline model for *R*_*0*_=1.5 and 2.5.

**Scenario D:** Increasing the case fatality rate by a factor of 10. This means increasing the severity of the pandemics to levels similar to those observed during the Spanish Flu [36]. In this case, the optimal strategies were closing kindergartens, primary and secondary schools for 9 weeks if *R*_*0*_=1.5, 7 weeks if *R*_*0*_=2.0, and 5 weeks if *R*_*0*_= 2.5 (Additional file 1: e-Table 3).

**Scenario E:** Decreasing the case fatality rate by a factor of 10. In this case, when *R*_*0*_=1.5, closing secondary school for 6 weeks is most cost-effective. Otherwise, there is no cost-effective strategy among the strategies we examined (Additional file 1: e-Table 4).

**Scenario F:** Pandemic with 2009 H1N1 characteristics. The results show that the added cost of school closure was higher than not closing schools, regardless of the age target of school children. Consequently school closure would not have been cost-effective during the 2009 H1N1 pandemic (Additional file 1: e-Table 5).

## Discussion

Our study shows that school closure during influenza pandemic has a moderate impact on the total disease burden. The cost-effectiveness of school closure varies considerably across different strategies with different target groups and durations. Generally we found that for *R*_*0*_=1.5, 2.0 and 2.5 pandemics with case fatality rates of 0.1-0.2%, only those strategies involving closure of secondary schools were cost-effective from a societal point of view. The study shows that optimal school closure depends on the transmissibility and severity of the pandemic and may provide guidance to local policy planning. The optimal duration of closing secondary schools is shorter (4 weeks) with *R*_*0*_=2.5 compared to 8 weeks with *R*_*0*_=1.5. In contrast, school closure involving primary schools and kindergartens incur substantial economic costs due to lost productivity of care-taking parents. Consequently, most school closure strategies cannot be considered cost-effective (Tables 3, 4 and 5) at current values of quality adjusted life-years in Norway [37]. However, school closure involving children in need of parental care may be indicated when case fatality rates are high, for instance in the event of a future pandemic with an avian (H5N1) virus.

We also simulated a pandemic with characteristics of the 2009 H1N1 pandemic. Our results suggest that school closure as a single intervention would not have been cost-effective during the recent pandemic. This finding is in agreement with results by Brown and co-workers [17], who found that the net costs of school closure during the 2009 H1N1 pandemic would have been substantially higher than the cost savings from preventing influenza disease. However, other studies indicate that school closure might have been cost-effective, despite the low severity and low transmissibility of the 2009 H1N1 pandemic. Halder and co-workers [16] found that short-duration school closure of 2 to 4 weeks would be relatively cost-effective while in general school closure intervention as a single strategy would be less efficient than strategies involving widespread use of antivirals, and Araz and co-workers found that a 0.5% prevalence closure trigger followed by a 12 week closure would be cost-effective [20].

Our findings are similar to other computer simulation studies [8-10,17,36] and a surveillance data study from Hong Kong [7], all of which indicate that the impact of school closure on the pandemic is modest. In general we found that school closure peak timing was delayed with only few days compared with that of an unmitigated pandemic. The delay increased with lower transmissibility. The maximum delay was observed for intermediate closure durations, when the epidemic re-started influenced by the higher transmissibility of the unmitigated pandemic (*R*_*eff*_ > 1). A micro-simulation study by Lee and co-workers [9] also show that intermediate duration closure produces the longest delays. However, their observed delay for long closure duration was longer: 4–8 days for system wide school closure for *R*_*0*_=1.4-2.4. One possible explanation for the shorter delay in our study is that we assume that the whole population is interacting, while we did not model the individual transmission processes. In addition, individuals in our model generally mix most with individuals in their own age group. Therefore, there is a tendency that the epidemic in school children develops “independently” of how the epidemic develops in the other age groups, and school closure has only small impact on the disease burden in the population that is not directly affected by the intervention. We have performed additional simulations using a lower closure trigger of 0.5% instead of the 1% assumed in the baseline scenario (results not shown). These simulations show that an earlier trigger increases the maximum delay by approximately one third, while the peak timing during long duration closure increased only little.

Our approach is analogous to a recent study by Araz and co-workers [20], using a dynamic compartmental model combined with calculations of incremental cost-effectiveness ratios to select the preferred policy. They studied pandemics with transmissibility in the range *R*_*0*_=1.1-2.1, using various closure triggers and fixed school closure durations of 1–24 weeks or prevalence-based reopening triggers. They found that in low transmissibility scenarios, early triggers combined with long closure duration of 12–24 weeks were preferred, regardless of severity; for high transmissibility scenarios, later triggers combined with 8–18 weeks closure were preferred. In comparison, our selected strategies involved much shorter closure durations of 4–8 weeks. One reason for this large discrepancy could be that they used early triggers. In addition, their model has a very long serial interval of 9 days, whereas our model has a serial interval of approximately 4 days due to the infectious profile, which we believe is more in agreement with data [38].

The present work highlights the potential importance of school closure among students who do not need parental care. The benefit of school closure interventions targeting this group appears to have escaped notice in the literature. Our results suggest that closing secondary school alone can decrease the peak prevalence of symptomatic infection by 10–20% while incurring no loss of productivity for parents. Hence, school closure for children over 12 years could have important implications for the functioning of the healthcare system during the surge of a pandemic, when the capacity of health services may be pressured. We note that in Norway laptop computers are mandatory equipment in secondary schools and an organized computer network (“Fronter”) for communication between students and teachers in primary and secondary schools is already in place. It would therefore be possible to plan for sustained teaching and learning during an extended school closure, making secondary school closure even more cost-effective. However, for the strategy to be effective, it is important that students actually follow the recommendations and isolate themselves. This may be difficult to achieve for extended periods of time.

The health-economic evaluation in this study was based on estimates of age-specific health-outcome from previous pandemics [26]. If we scale up the results in the baseline scenarios for *R*_*0*_=1.5-2.5 pandemics to the national level (Oslo comprises approximately 12% of Norwegian population), our results correspond to 16 000–26 000 hospitalizations and 4 900–8 200 deaths in Norway with an attack rate ranging from 37-58%. In comparison, the yearly influenza epidemics (attack rate of 5-10%) results in approximately 2 700 cases of hospitalizations [34] and approximately 1 000 deaths [39]. Adjusting for the difference in attack rates, this indicates that our results are in reasonable agreement with findings from the seasonal epidemics; however, the numbers are difficult to compare because the seasonal epidemics primarily affect the elderly population.

Our study has several limitations. Firstly, the age-specific contact rate data were adopted from a Dutch study, as no Norwegian data on social mixing is currently available. The contact pattern in Norway may differ, in particular due to the high attendance rates in kindergarten and high employment rate of women. Secondly, the effect of school closure on the contact pattern in the population is not well documented in the literature and is uncertain. However, our choices were guided by observation from weekends and holidays and previous school closures in Oslo due to strikes, etc. Thirdly, the cost of lost learning is uncertain. We used tuition fees as a proxy for the value of learning, but private schools are primarily used by people with higher incomes and the tuition fee may therefore overstate the value of lost learning. Fourthly, productivity losses may be overestimated because some parents who are away from work may be absent anyway because they have influenza themselves. Fifthly, energy savings in schools during school closure may be partly off-set by higher energy use in homes. However, energy in Norway is cheap and only small proportions of households have day-time energy saving systems according to the governmental energy saving organization. Lastly, we have considered school closure as a single strategy. Combining school closure with other interventions such as use of antiviral medications or other social distancing measures might change the conclusions about optimal duration of school closure, and the target group.

## Conclusions

School closure has moderate impact on influenza disease and may incur substantial economic costs in terms of lost productivity from care-taking parents absent from work. Closing secondary schools, assuming children above 12 years would not need parental care, is a cost-effective strategy from a societal perspective. With the current willingness to pay in Norway, closing kindergartens and primary schools is not a cost-effective policy to mitigate an influenza pandemic, unless the case fatality rates are high. Reliable information on influenza mortality is therefore of primary importance to inform decision-making on school closure. Finally, we note that the perspective of the policy maker is crucial for optimal design of school closure. If the policy maker disregards productivity losses, the optimal strategy is to close as many school as possible for as long time as possible.

## Competing interests

The authors declare that they have no competing interest.

## Authors’ contributions

YX originated the idea and drafted the paper. YX and BFB constructed the mathematical model while YX and ISK conducted the health economic evaluation. BFB and ISK reviewed and revised the manuscript. All authors read and approved the final manuscript.

## Funding source

Yiting Xue was supported by the Norwegian Research Council through project number 177401/V50 and Birgitte Freiesleben de Blasio was supported by the Norwegian Research Council through project number 166056/V50.

## Pre-publication history

The pre-publication history for this paper can be accessed here:

http://www.biomedcentral.com/1471-2458/12/962/prepub

## Supplementary Material

Additional file 1 Online_additional_materials.doc, 1233K. Click here for file

## References

[B1] Cox NJ Fukuda K Influenza Infect Dis Clin North Am 1998 12 27 38 10.1016/S0891-5520(05)70406-2 9494827

[B2] Yang Y Sugimoto JD Halloran ME Basta NE Chao DL Matrajt L The transmissibility and control of pandemic influenza A (H1N1) virus Science 2009 326 729 733 10.1126/science.1177373 19745114 PMC2880578

[B3] Cauchemez S Ferguson NM Wachtel C Tegnell A Saour G Duncan B Closure of schools during an influenza pandemic Lancet Infect Dis 2009 9 473 481 10.1016/S1473-3099(09)70176-8 19628172 PMC7106429

[B4] Hatchett RJ Mecher CE Lipsitch M Public health interventions and epidemic intensity during the 1918 influenza pandemic Proc Natl Acad Sci U S A 2007 104 7582 7587 10.1073/pnas.0610941104 17416679 PMC1849867

[B5] Markel H Lipman HB Navarro JA Sloan A Michalsen JR Stern AM Nonpharmaceutical interventions implemented by US cities during the 1918–1919 influenza pandemic JAMA 2007 298 644 654 10.1001/jama.298.6.644 17684187

[B6] Hens N Ayele GM Goeyvaerts N Aerts M Mossong J Edmunds JW Estimating the impact of school closure on social mixing behaviour and the transmission of close contact infections in eight European countries BMC Infect Dis 2009 9 187 10.1186/1471-2334-9-187 19943919 PMC2799408

[B7] Cowling BJ Lau EH Lam CL Cheng CK Kovar J Chan KH Effects of school closures, 2008 winter influenza season, Hong Kong Emerg Infect Dis 2008 14 1660 1662 10.3201/eid1410.080646 18826841 PMC2609897

[B8] Glass K Barnes B How much would closing schools reduce transmission during an influenza pandemic? Epidemiology 2007 18 623 628 10.1097/EDE.0b013e31812713b4 17700251

[B9] Lee BY Brown ST Cooley P Potter MA Wheaton WD Voorhees RE Simulating School Closure Strategies to Mitigate an Influenza Epidemic J Public Health Manag Pract 2010 16 3 252 61 20035236 10.1097/PHH.0b013e3181ce594e PMC2901099

[B10] Glass RJ Glass LM Beyeler WE Min HJ Targeted social distancing design for pandemic influenza Emerg Infect Dis 2006 12 1671 1681 10.3201/eid1211.060255 17283616 PMC3372334

[B11] Halder N Kelso JK Milne GJ Analysis of the effectiveness of interventions used during the 2009 A/H1N1 influenza pandemic BMC Publ Health 2010 10 168 10.1186/1471-2458-10-168 PMC2853510 20346187

[B12] Halder N Kelso JK Milne GJ Developing guidelines for school closure interventions to be used during a future influenza pandemic BMC Infect Dis 2010 10 221 10.1186/1471-2334-10-221 20659348 PMC2915996

[B13] Sadique MZ Adams EJ Edmunds WJ Estimating the costs of school closure for mitigating an influenza pandemic BMC Publ Health 2008 8 135 10.1186/1471-2458-8-135 PMC2377259 18435855

[B14] Lempel H Epstein JM Hammond RA Economic cost and health care workforce effects of school closures in the U.S, RRN1051 PLoS Curr Influenza 2009 10.1371/currents.RRN1051 PMC2762813 20025205

[B15] House T Baguelin M Van Hoek AJ White PJ Sadique Z Eames K Modelling the impact of local reactive school closures on critical care provision during an influenza pandemic Proc Biol Sci 2011 278 2753 2760 10.1098/rspb.2010.2688 21288945 PMC3145187

[B16] Halder N Kelso JK Milne GJ Cost-effective strategies for mitigating a future influenza pandemic with H1N1 2009 characteristics PLoS One 2011 6 e22087 10.1371/journal.pone.0022087 21760957 PMC3132288

[B17] Brown ST Tai JH Bailey RR Cooley PC Wheaton WD Potter MA Would school closure for the 2009 H1N1 influenza epidemic have been worth the cost? A computational simulation of Pennsylvania BMC Publ Health 2011 11 353 10.1186/1471-2458-11-353 PMC3119163 21599920

[B18] Perlroth DJ Glass RJ Davey VJ Cannon D Garber AM Owens DK Health Outcomes and Costs of Community Mitigation Strategies for an Influenza Pandemic in the United States Clin Infect Dis 2010 50 165 174 10.1086/649867 20021259

[B19] Sander B Nizam A Garrison LP Postma MJ Halloran ME Longini IM Economic evaluation of influenza pandemic mitigation strategies in the US using a stochastic microsimulation influenza model Value Health 2007 10 A19 10.1111/j.1524-4733.2008.00437.x PMC3710126 18671770

[B20] Araz OM Damien P Paltiel DA Burke S Geijn BV Galvani A Simulating school closure policies for cost effective pandemic decision making BMC Publ Health 2012 12 449 10.1186/1471-2458-12-449 PMC3495022 22713694

[B21] Statistics Norwayhttp://www.ssb.no/. 16-12-2010.

[B22] Keeling MJ Rohani P Modeling Infectious Diseases in Humans and Animals 2007

[B23] Carrat F Vergu E Ferguson NM Lemaitre M Cauchemez S Leach S Time lines of infection and disease in human influenza: a review of volunteer challenge studies Am J Epidemiol 2008 167 775 785 10.1093/aje/kwm375 18230677

[B24] Longini IM Halloran ME Nizam A Yang Y Containing pandemic influenza with antiviral agents Am J Epidemiol 2004 159 623 633 10.1093/aje/kwh092 15033640

[B25] Germann TC Kadau K Longini IM Jr Macken CA Mitigation strategies for pandemic influenza in the United States Proc Natl Acad Sci U S A 2006 103 5935 5940 10.1073/pnas.0601266103 16585506 PMC1458676

[B26] Fraser C Riley S Anderson RM Ferguson NM Factors that make an infectious disease outbreak controllable Proc Natl Acad Sci U S A 2004 101 6146 6151 10.1073/pnas.0307506101 15071187 PMC395937

[B27] Fraser C Donnelly CA Cauchemez S Hanage WP Van Kerkhove MD Hollingsworth TD Pandemic potential of a strain of influenza A (H1N1): early findings Science 2009 324 1557 1561 10.1126/science.1176062 19433588 PMC3735127

[B28] Lau LL Cowling BJ Fang VJ Chan KH Lau EH Lipsitch M Viral shedding and clinical illness in naturally acquired influenza virus infections J Infect Dis 2010 201 1509 1516 10.1086/652241 20377412 PMC3060408

[B29] Wallinga J Teunis P Kretzschmar M Using data on social contacts to estimate age-specific transmission parameters for respiratory-spread infectious agents Am J Epidemiol 2006 164 936 944 10.1093/aje/kwj317 16968863

[B30] Gani R Hughes H Fleming D Griffin T Medlock J Leach S Potential impact of antiviral drug use during influenza pandemic Emerg Infect Dis 2005 11 1355 1362 10.3201/eid1209.041344 16229762 PMC3371825

[B31] Comay Y Melnik A Pollatschek MA The Option Value of Education and the Optimal Path for Investment in Human Capital Int Econ Rev 1973 14 421 435 10.2307/2525931

[B32] Maurin E McNally S Vive la revolution! Long-term educational returns of 1968 to the angry students J Labor Econ 2008 26 1 33 10.1086/522071

[B33] Meltzer MI Cox NJ Fukuda K The economic impact of pandemic influenza in the United States: priorities for intervention Emerg Infect Dis 1999 5 659 671 10.3201/eid0505.990507 10511522 PMC2627723

[B34] Xue Y Kristiansen IS de Blasio BF Modeling the cost of influenza: the impact of missing costs of unreported complications and sick leave BMC Publ Health 2010 10 724 10.1186/1471-2458-10-724 PMC3009644 21106057

[B35] O'Brien BJ Goeree R Blackhouse G Smieja M Loeb M Oseltamivir for treatment of influenza in healthy adults: pooled trial evidence and cost-effectiveness model for Canada Value Health 2003 6 116 125 10.1046/j.1524-4733.2003.00213.x 12641862

[B36] Cauchemez S Valleron AJ Boelle PY Flahault A Ferguson NM Estimating the impact of school closure on influenza transmission from Sentinel data Nature 2008 452 750 754 10.1038/nature06732 18401408

[B37] The Norwegian Directorate of Health Health effects in social-economics analysis (Helseeffekter i samfunnsøkonomiske analyser) 2007 http://www.helsedirektoratet.no/publikasjoner/helseeffekter-i-samfunnsokonomiske-analyser-/Sider/default.aspx.

[B38] Boelle PY Ansart S Cori A Valleron AJ Transmission parameters of the A/H1N1 (2009) influenza virus pandemic: a review Influenza Other Respi Viruses 2011 5 306 316 10.1111/j.1750-2659.2011.00234.x 21668690 PMC4942041

[B39] Gran JM Iversen B Hungnes O Aalen OO Estimating influenza-related excess mortality and reproduction numbers for seasonal influenza in Norway, 1975–2004 Epidemiol Infect 2010 138 1559 1568 10.1017/S0950268810000671 20334732

