# Supplementary material for: Dynamic modelling of costs and health consequences of school closure during an influenza pandemic
Source: BMC Public Health. 2012 Nov 9;12:962. doi: 10.1186/1471-2458-12-962 (PMC3533523; doi:10.1186/1471-2458-12-962)
Supplement: Additional file 1 — Online_additional_materials.doc, 1233K. [file 1471-2458-12-962-S1.doc]

**e-Figure 1: Cost-effectiveness acceptability curve when secondary schools are closed for 8 weeks using Monte Carlo simulation (n=1000)**


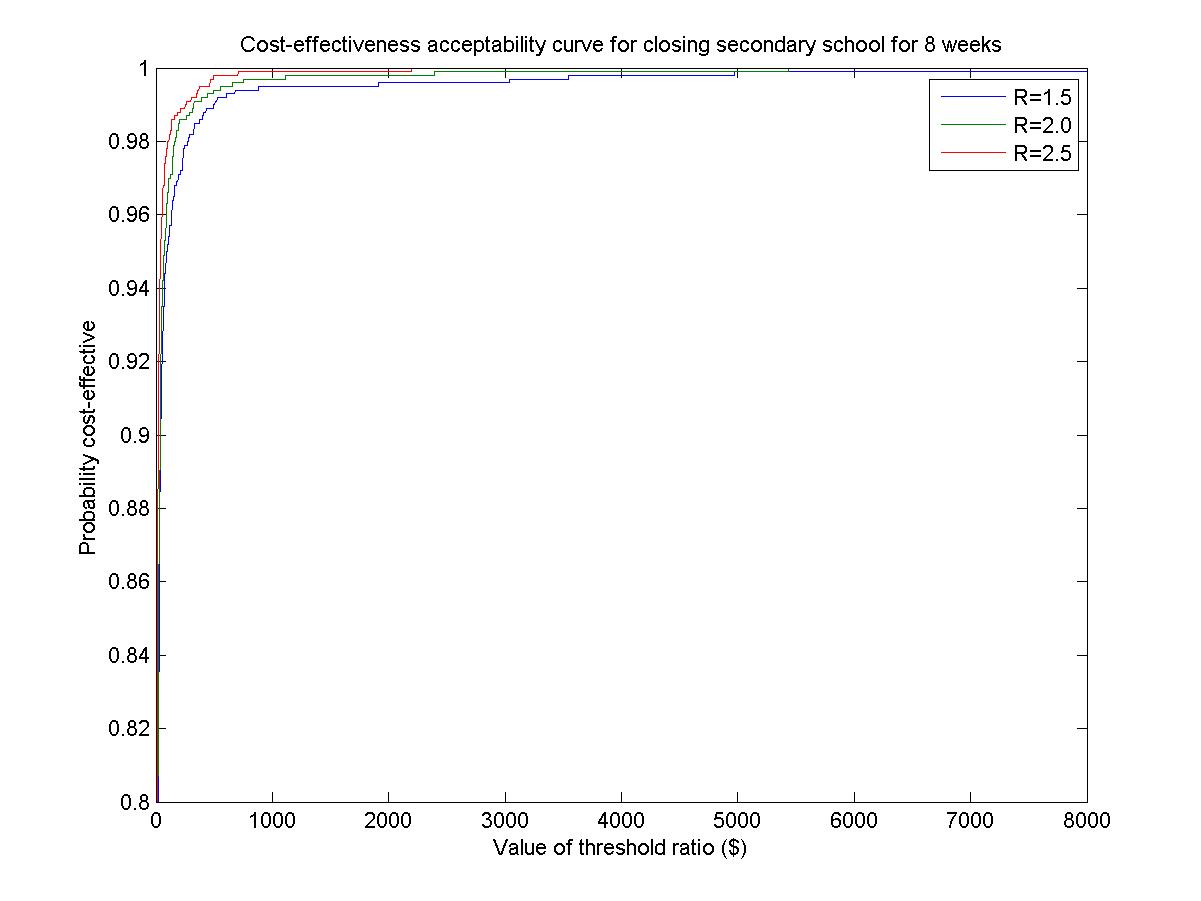


**e-Figure 2: Epidemic curves for $R_{0}$= 1.5, 2.0 and 2.5 with varying duration of school closure. Simulations are performed closing kindergarten, primary and secondary schools.**

**e-Figure 2(A): Basic reproductive number$R_{0}$ =1.5.**

**
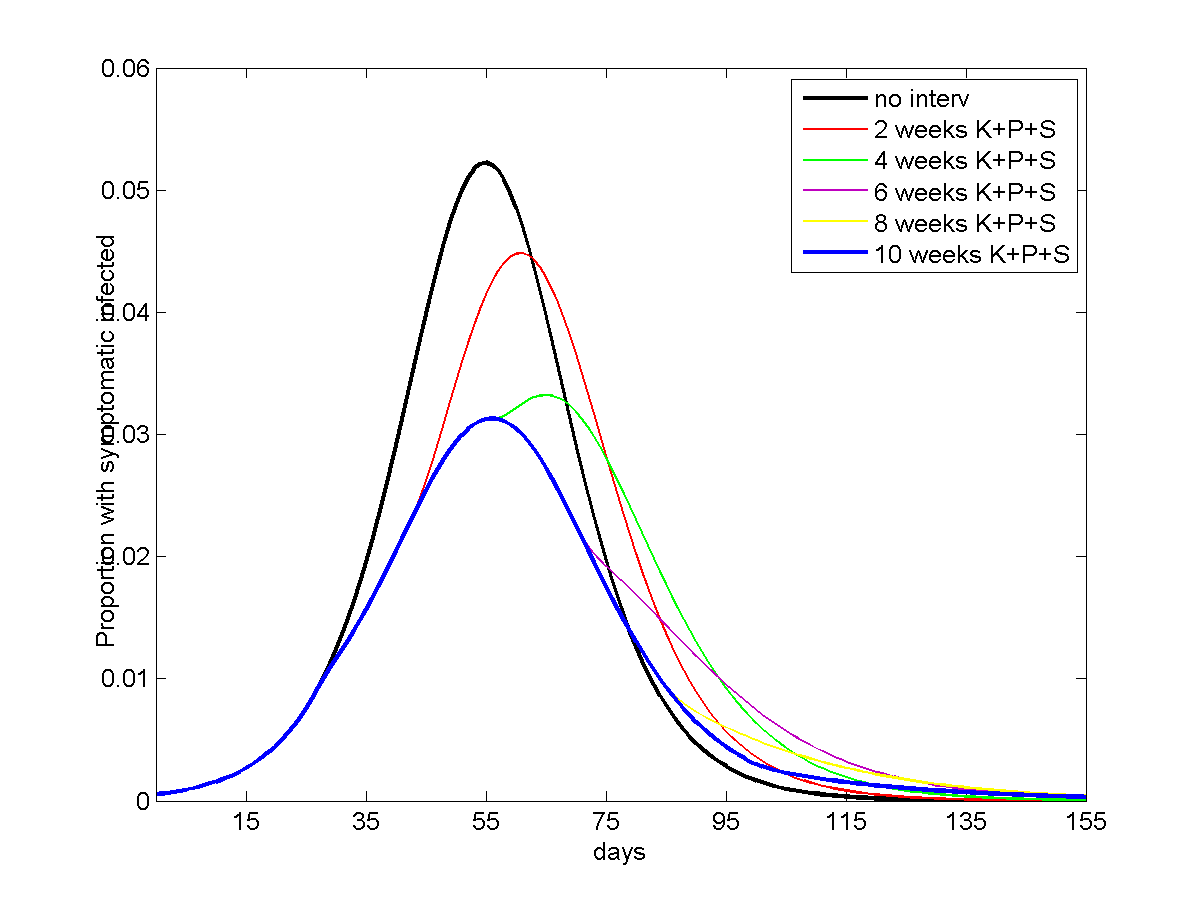
**

**e-Figure 2(B): Basic reproductive number$R_{0}$ =2.0.**

**
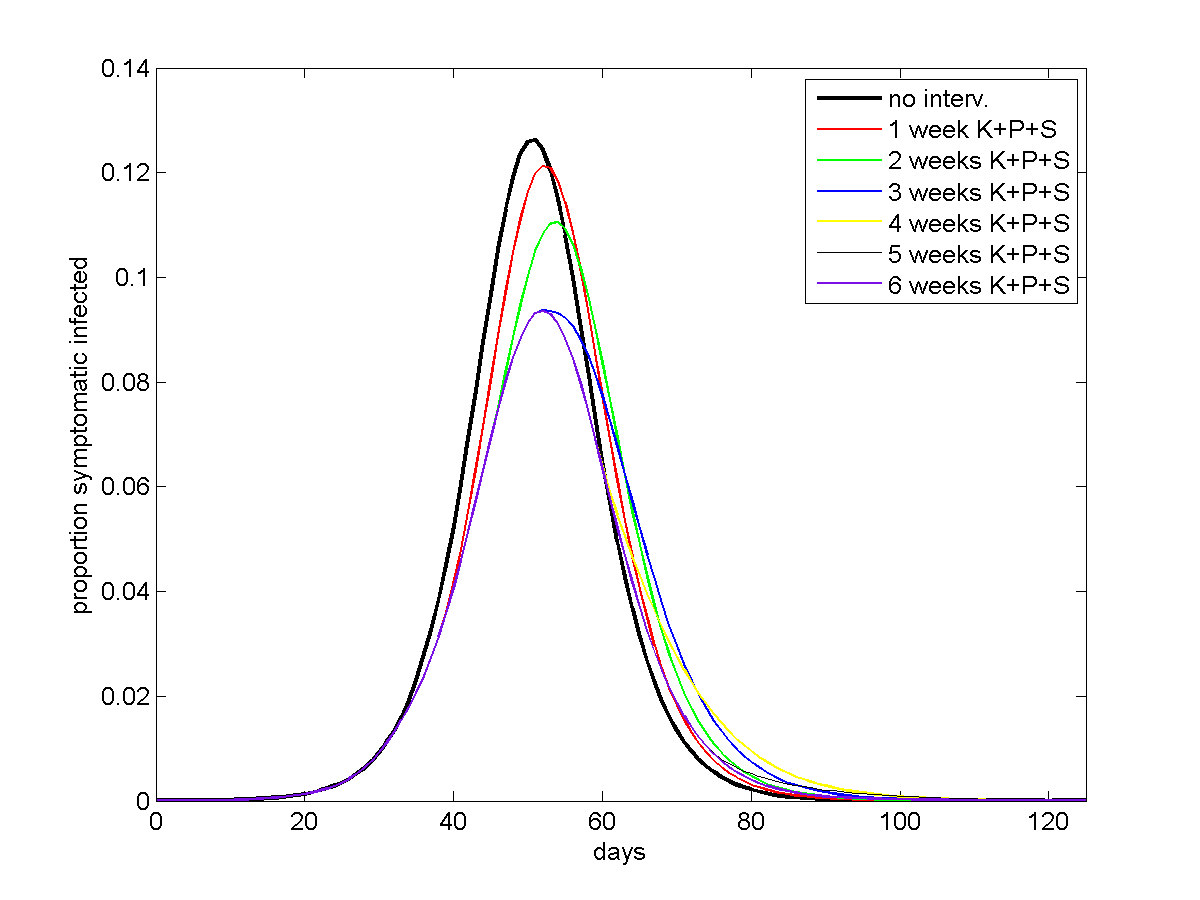
**

**e-Figure 2(C): Basic reproductive number$R_{0}$ = 2.5**

**
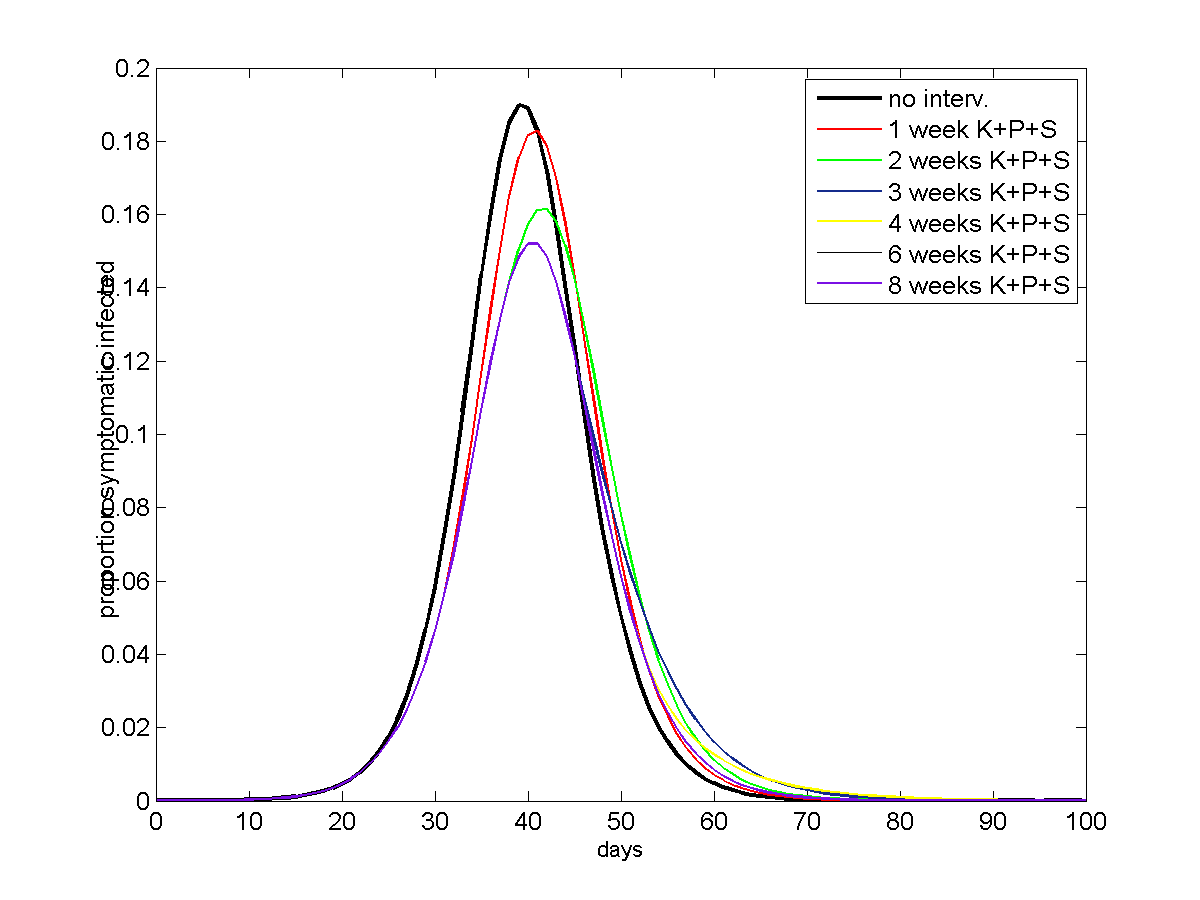
**

**e-Table 1 (Scenario B): Cost and health outcome according to type and duration of school closure (The most cost-effective option is highlighted)**

Note: the maximum willingness to pay is set to be NOK 500,000 or US$71,500 based on the government guidance ^28^

**e-Table 1 I: *R_0_***=1.5

| **Target school** | **Duration (weeks)** | **Cost of lost learning ($1000)** | **Lost productivity due to school closure ($1000)** | **Energy savings ($1000)** | **Health care costs ($1000)** | **Lost productivity due to fatal cases ($1000)** | **Lost productivity due to sickness ($1000)** | **Cost of Oslo municipality ($1000)** | **Total cost ($1000)** | **QALY loss** | **QALY gain (relative)** | **ICER (compared to no intervention) ($ per QALY** | **ICER (relative)** |
| --- | --- | --- | --- | --- | --- | --- | --- | --- | --- | --- | --- | --- | --- |
| 0 | 0 | 0 | 0 | 0 | 20 591 | 312 958 | 101 576 | 20 591 | 435 125 | 10 591 | 0 |  |  |
| 3 | 6 | 19 350 | 0 | 1 080 | 19 557 | 298 239 | 97 846 | 18 477 | 433 912 | 10 084 | 507 | -2 395 |  |
| 3 | 7 | 22 575 | 0 | 1 260 | 19 410 | 296 139 | 97 312 | 18 150 | 434 175 | 10 012 | 579 | -1 641 | 3 648 |
| 3 | 5 | 16 125 | 0 | 900 | 19 766 | 301 213 | 98 600 | 18 866 | 434 804 | 10 187 | 404 | -796 | Dominated |
| 3 | 8 | 25 800 | 0 | 1 440 | 19 318 | 294 825 | 96 978 | 17 878 | 435 481 | 9 967 | 624 | 570 | 28 929 |
| 3 | 4 | 12 900 | 0 | 720 | 20 008 | 304 661 | 99 474 | 19 288 | 436 323 | 10 305 | 286 | 4 193 | Dominated |
| 3 | 1 | 3 225 | 0 | 180 | 20 509 | 311 792 | 101 278 | 20 329 | 436 625 | 10 551 | 40 | 37 316 | Dominated |
| 3 | 9 | 29 025 | 0 | 1 620 | 19 264 | 294 064 | 96 784 | 17 644 | 437 517 | 9 941 | 650 | 3 679 | 77 819 |
| 3 | 3 | 9 675 | 0 | 540 | 20 235 | 307 897 | 100 293 | 19 695 | 437 560 | 10 417 | 174 | 13 962 | Dominated |
| 3 | 2 | 6 450 | 0 | 360 | 20 403 | 310 287 | 100 897 | 20 043 | 437 678 | 10 499 | 92 | 27 727 | Dominated |
| 3 | 10 | 32 250 | 0 | 1 800 | 19 237 | 293 672 | 96 684 | 17 437 | 440 043 | 9 928 | 664 | 7 412 | 187 991 |
| 2 | 1 | 0 | 26 795 | 188 | 20 452 | 310 917 | 101 014 | 20 264 | 458 991 | 10 522 | 69 | 347 424 |  |
| 1 | 1 | 0 | 36 194 | 174 | 20 500 | 311 616 | 101 203 | 20 326 | 469 339 | 10 546 | 45 | 758 050 |  |
| 2 | 2 | 0 | 53 591 | 376 | 20 281 | 308 370 | 100 299 | 19 905 | 482 164 | 10 437 | 154 | 305 521 |  |
| 4 | 1 | 0 | 62 989 | 362 | 20 366 | 309 645 | 100 659 | 20 004 | 493 298 | 10 480 | 111 | 522 031 |  |
| 5 | 1 | 3 225 | 62 989 | 542 | 20 294 | 308 620 | 100 395 | 19 752 | 494 982 | 10 444 | 147 | 407 701 |  |
| 1 | 2 | 0 | 72 388 | 348 | 20 374 | 309 754 | 100 683 | 20 026 | 502 851 | 10 483 | 108 | 629 589 |  |
| 2 | 3 | 0 | 80 386 | 564 | 20 022 | 304 541 | 99 232 | 19 458 | 503 617 | 10 309 | 282 | 242 584 |  |
| 2 | 4 | 0 | 107 181 | 752 | 19 680 | 299 501 | 97 844 | 18 928 | 523 455 | 10 139 | 452 | 195 499 |  |
| 1 | 3 | 0 | 108 582 | 522 | 20 181 | 306 890 | 99 886 | 19 659 | 535 016 | 10 387 | 204 | 490 288 |  |
| 2 | 5 | 0 | 133 976 | 940 | 19 270 | 293 486 | 96 208 | 18 330 | 542 000 | 9 936 | 655 | 163 224 |  |
| 4 | 2 | 0 | 125 979 | 724 | 20 103 | 305 737 | 99 558 | 19 379 | 550 653 | 10 349 | 242 | 476 798 |  |
| 5 | 2 | 6 450 | 125 979 | 1 084 | 19 970 | 303 854 | 99 080 | 18 886 | 554 249 | 10 284 | 308 | 387 378 |  |
| 2 | 6 | 0 | 160 772 | 1 128 | 18 876 | 287 740 | 94 664 | 17 748 | 560 924 | 9 742 | 849 | 148 135 |  |
| 1 | 4 | 0 | 144 776 | 696 | 19 927 | 303 155 | 98 853 | 19 231 | 566 015 | 10 262 | 329 | 397 316 |  |
| 2 | 7 | 0 | 187 567 | 1 316 | 18 546 | 282 943 | 93 387 | 17 230 | 581 127 | 9 579 | 1 012 | 144 278 |  |
| 1 | 5 | 0 | 180 970 | 870 | 19 659 | 299 210 | 97 771 | 18 789 | 596 740 | 10 129 | 463 | 349 426 |  |
| 2 | 8 | 0 | 214 362 | 1 504 | 18 312 | 279 543 | 92 487 | 16 808 | 603 200 | 9 464 | 1 127 | 149 073 |  |
| 4 | 3 | 0 | 188 968 | 1 086 | 19 724 | 300 109 | 97 977 | 18 638 | 605 692 | 10 160 | 431 | 395 760 |  |
| 5 | 3 | 9 675 | 188 968 | 1 626 | 19 529 | 297 361 | 97 287 | 17 903 | 611 194 | 10 064 | 527 | 334 378 |  |
| 2 | 9 | 0 | 241 157 | 1 692 | 18 164 | 277 406 | 91 923 | 16 472 | 626 958 | 9 391 | 1 200 | 159 841 |  |
| 1 | 6 | 0 | 217 164 | 1 044 | 19 433 | 295 909 | 96 871 | 18 389 | 628 334 | 10 017 | 574 | 336 521 |  |
| 2 | 10 | 0 | 267 953 | 1 880 | 18 076 | 276 130 | 91 587 | 16 196 | 651 865 | 9 347 | 1 244 | 174 287 |  |
| 4 | 4 | 0 | 251 957 | 1 448 | 19 221 | 292 662 | 95 895 | 17 773 | 658 287 | 9 910 | 681 | 327 605 |  |
| 1 | 7 | 0 | 253 358 | 1 218 | 19 275 | 293 596 | 96 244 | 18 057 | 661 255 | 9 939 | 652 | 346 570 |  |
| 5 | 4 | 12 900 | 251 957 | 2 168 | 18 958 | 288 973 | 94 970 | 16 790 | 665 590 | 9 781 | 810 | 284 585 |  |
| 1 | 8 | 0 | 289 552 | 1 392 | 19 175 | 292 142 | 95 852 | 17 783 | 695 329 | 9 889 | 702 | 370 782 |  |
| 4 | 5 | 0 | 314 946 | 1 810 | 18 627 | 283 914 | 93 461 | 16 817 | 709 138 | 9 615 | 976 | 280 802 |  |
| 5 | 5 | 16 125 | 314 946 | 2 710 | 18 292 | 279 195 | 92 265 | 15 582 | 718 113 | 9 451 | 1 140 | 248 143 |  |
| 1 | 9 | 0 | 325 746 | 1 566 | 19 120 | 291 343 | 95 636 | 17 554 | 730 280 | 9 862 | 729 | 404 946 |  |
| 4 | 6 | 0 | 377 936 | 2 172 | 18 020 | 274 973 | 90 982 | 15 848 | 759 739 | 9 313 | 1 278 | 254 079 |  |
| 1 | 10 | 0 | 361 940 | 1 740 | 19 093 | 290 949 | 95 530 | 17 353 | 765 772 | 9 849 | 742 | 445 452 |  |
| 5 | 6 | 19 350 | 377 936 | 3 252 | 17 593 | 268 925 | 89 414 | 14 341 | 769 965 | 9 103 | 1 488 | 225 028 |  |
| 4 | 7 | 0 | 440 925 | 2 534 | 17 472 | 266 929 | 88 755 | 14 938 | 811 547 | 9 041 | 1 550 | 242 908 |  |
| 5 | 7 | 22 575 | 440 925 | 3 794 | 16 897 | 258 712 | 86 566 | 13 103 | 821 881 | 8 757 | 1 834 | 210 895 |  |
| 4 | 8 | 0 | 503 914 | 2 896 | 17 042 | 260 623 | 87 009 | 14 146 | 865 692 | 8 828 | 1 763 | 244 200 |  |
| 5 | 8 | 25 800 | 503 914 | 4 336 | 16 247 | 249 150 | 83 882 | 11 911 | 874 658 | 8 433 | 2 158 | 203 679 |  |
| 4 | 9 | 0 | 566 903 | 3 258 | 16 731 | 256 063 | 85 746 | 13 473 | 922 185 | 8 673 | 1 918 | 253 981 |  |
| 5 | 9 | 29 025 | 566 903 | 4 878 | 15 727 | 241 496 | 81 721 | 10 849 | 929 995 | 8 174 | 2 417 | 204 704 |  |
| 4 | 10 | 0 | 629 893 | 3 620 | 16 521 | 252 989 | 84 893 | 12 901 | 980 675 | 8 569 | 2 022 | 269 816 |  |
| 5 | 10 | 32 250 | 629 893 | 5 420 | 15 295 | 235 133 | 79 912 | 9 875 | 987 063 | 7 958 | 2 633 | 209 602 |  |

**e-Table 1 II: *R_0_*=2.0**

| **Target school** | **Duration (weeks)** | **Cost of lost learning** | **Lost productivity due to school closure** | **Energy savings** | **Health care costs** | **Lost productivity due to fatal cases** | **Lost productivity due to sickness** | **Cost of Oslo municipality** | **Total cost** | **QALY loss** | **QALY gain(compared with no intervention)** | **ICER (compared to no intervention) ($ per QALY** | **ICER (relative)** |
| --- | --- | --- | --- | --- | --- | --- | --- | --- | --- | --- | --- | --- | --- |
| 0 | 0 | 0 | 0 | 0 | 28 890 | 428 137 | 138 654 | 28 890 | 595 682 | 14 912 |  |  |  |
| 3 | 4 | 12 900 | 0 | 720 | 28 215 | 419 135 | 136 843 | 27 495 | 596 374 | 14 591 | 321 | 2 155 |  |
| 3 | 5 | 16 125 | 0 | 900 | 28 049 | 416 920 | 136 411 | 27 149 | 596 604 | 14 512 | 400 | 2 306 | 2 921 |
| 3 | 1 | 3 225 | 0 | 180 | 28 846 | 427 542 | 138 529 | 28 666 | 597 961 | 14 891 | 21 | 106 854 |  |
| 3 | 3 | 9 675 | 0 | 540 | 28 491 | 422 813 | 137 570 | 27 951 | 598 009 | 14 722 | 190 | 12 224 | Dominated |
| 3 | 6 | 19 350 | 0 | 1 080 | 27 985 | 416 062 | 136 245 | 26 905 | 598 562 | 14 482 | 431 | 6 686 | 64 224 |
| 3 | 2 | 6 450 | 0 | 360 | 28 732 | 426 018 | 138 216 | 28 372 | 599 056 | 14 837 | 76 | 44 470 | Dominated |
| 3 | 7 | 22 575 | 0 | 1 260 | 27 964 | 415 780 | 136 190 | 26 704 | 601 248 | 14 472 | 441 | 12 628 | 267 404 |
| 3 | 8 | 25 800 | 0 | 1 440 | 27 957 | 415 695 | 136 173 | 26 517 | 604 186 | 14 469 | 444 | 19 161 | 975 711 |
| 3 | 9 | 29 025 | 0 | 1 620 | 27 955 | 415 672 | 136 169 | 26 335 | 607 201 | 14 468 | 445 | 25 907 | 3 654 485 |
| 3 | 10 | 32 250 | 0 | 1 800 | 27 955 | 415 664 | 136 167 | 26 155 | 610 236 | 14 468 | 445 | 32 714 | 11 358 909 |
| 2 | 1 | 0 | 26 795 | 188 | 28 820 | 427 127 | 138 398 | 28 632 | 620 953 | 14 878 | 34 | 736 022 |  |
| 1 | 1 | 0 | 36 194 | 174 | 28 835 | 427 354 | 138 465 | 28 661 | 630 675 | 14 886 | 27 | 1 307 627 |  |
| 2 | 2 | 0 | 53 591 | 376 | 28 650 | 424 657 | 137 762 | 28 274 | 644 283 | 14 795 | 118 | 412 865 |  |
| 4 | 1 | 0 | 62 989 | 362 | 28 769 | 426 399 | 138 221 | 28 407 | 656 017 | 14 853 | 59 | 1 018 685 |  |
| 5 | 1 | 3 225 | 62 989 | 542 | 28 729 | 425 858 | 138 105 | 28 187 | 658 364 | 14 834 | 79 | 796 756 |  |
| 1 | 2 | 0 | 72 388 | 348 | 28 691 | 425 301 | 137 970 | 28 343 | 664 002 | 14 816 | 97 | 705 679 |  |
| 2 | 3 | 0 | 80 386 | 564 | 28 294 | 419 588 | 136 527 | 27 730 | 664 231 | 14 622 | 290 | 236 005 |  |
| 2 | 4 | 0 | 107 181 | 752 | 27 822 | 413 036 | 135 069 | 27 070 | 682 357 | 14 396 | 517 | 167 697 |  |
| 1 | 3 | 0 | 108 582 | 522 | 28 394 | 421 131 | 137 004 | 27 872 | 694 589 | 14 672 | 240 | 411 717 |  |
| 2 | 5 | 0 | 133 976 | 940 | 27 443 | 407 873 | 134 017 | 26 503 | 702 369 | 14 215 | 697 | 153 022 |  |
| 4 | 2 | 0 | 125 979 | 724 | 28 484 | 422 294 | 137 183 | 27 760 | 713 216 | 14 714 | 198 | 592 702 |  |
| 5 | 2 | 6 450 | 125 979 | 1 084 | 28 382 | 420 955 | 136 911 | 27 298 | 717 593 | 14 665 | 247 | 493 571 |  |
| 1 | 4 | 0 | 144 776 | 696 | 28 063 | 416 581 | 136 011 | 27 367 | 724 736 | 14 514 | 398 | 323 908 |  |
| 2 | 6 | 0 | 160 772 | 1 128 | 27 236 | 405 096 | 133 479 | 26 108 | 725 454 | 14 118 | 795 | 163 287 |  |
| 2 | 7 | 0 | 187 567 | 1 316 | 27 156 | 404 021 | 133 275 | 25 840 | 750 703 | 14 080 | 833 | 186 196 |  |
| 1 | 5 | 0 | 180 970 | 870 | 27 853 | 413 720 | 135 420 | 26 983 | 757 093 | 14 414 | 499 | 323 630 |  |
| 4 | 3 | 0 | 188 968 | 1 086 | 27 906 | 414 060 | 135 177 | 26 820 | 765 024 | 14 433 | 479 | 353 342 |  |
| 5 | 3 | 9 675 | 188 968 | 1 626 | 27 680 | 411 082 | 134 567 | 26 054 | 770 346 | 14 325 | 587 | 297 428 |  |
| 2 | 8 | 0 | 214 362 | 1 504 | 27 130 | 403 679 | 133 211 | 25 626 | 776 878 | 14 068 | 845 | 214 525 |  |
| 1 | 6 | 0 | 217 164 | 1 044 | 27 765 | 412 542 | 135 183 | 26 721 | 791 611 | 14 372 | 540 | 362 652 |  |
| 2 | 9 | 0 | 241 157 | 1 692 | 27 122 | 403 567 | 133 190 | 25 430 | 803 344 | 14 064 | 849 | 244 722 |  |
| 4 | 4 | 0 | 251 957 | 1 448 | 27 139 | 403 335 | 132 713 | 25 691 | 813 696 | 14 063 | 849 | 256 769 |  |
| 5 | 4 | 12 900 | 251 957 | 2 168 | 26 699 | 397 421 | 131 438 | 24 531 | 818 246 | 13 852 | 1 061 | 209 819 |  |
| 1 | 7 | 0 | 253 358 | 1 218 | 27 739 | 412 195 | 135 114 | 26 521 | 827 188 | 14 360 | 553 | 418 992 |  |
| 2 | 10 | 0 | 267 953 | 1 880 | 27 119 | 403 530 | 133 183 | 25 239 | 829 905 | 14 063 | 850 | 275 594 |  |
| 1 | 8 | 0 | 289 552 | 1 392 | 27 731 | 412 080 | 135 092 | 26 339 | 863 062 | 14 356 | 557 | 480 384 |  |
| 4 | 5 | 0 | 314 946 | 1 810 | 26 436 | 393 673 | 130 620 | 24 626 | 863 866 | 13 727 | 1 185 | 226 266 |  |
| 5 | 5 | 16 125 | 314 946 | 2 710 | 25 743 | 384 240 | 128 514 | 23 033 | 866 858 | 13 393 | 1 520 | 178 427 |  |
| 1 | 9 | 0 | 325 746 | 1 566 | 27 729 | 412 051 | 135 086 | 26 163 | 899 045 | 14 355 | 558 | 544 028 |  |
| 4 | 6 | 0 | 377 936 | 2 172 | 26 030 | 388 143 | 129 467 | 23 858 | 919 403 | 13 534 | 1 379 | 234 807 |  |
| 5 | 6 | 19 350 | 377 936 | 3 252 | 25 090 | 375 279 | 126 563 | 21 838 | 920 966 | 13 080 | 1 833 | 177 475 |  |
| 1 | 10 | 0 | 361 940 | 1 740 | 27 728 | 412 042 | 135 084 | 25 988 | 935 055 | 14 355 | 558 | 608 285 |  |
| 4 | 7 | 0 | 440 925 | 2 534 | 25 861 | 385 848 | 128 996 | 23 327 | 979 096 | 13 453 | 1 459 | 262 774 |  |
| 5 | 7 | 22 575 | 440 925 | 3 794 | 24 754 | 370 684 | 125 572 | 20 960 | 980 716 | 12 919 | 1 994 | 193 135 |  |
| 4 | 8 | 0 | 503 914 | 2 896 | 25 798 | 384 987 | 128 821 | 22 902 | 1 040 624 | 13 423 | 1 489 | 298 765 |  |
| 5 | 8 | 25 800 | 503 914 | 4 336 | 24 616 | 368 789 | 125 165 | 20 280 | 1 043 948 | 12 853 | 2 060 | 217 614 |  |
| 4 | 9 | 0 | 566 903 | 3 258 | 25 776 | 384 689 | 128 760 | 22 518 | 1 102 870 | 13 413 | 1 500 | 338 181 |  |
| 5 | 9 | 29 025 | 566 903 | 4 878 | 24 563 | 368 073 | 125 012 | 19 685 | 1 108 698 | 12 827 | 2 085 | 246 052 |  |
| 4 | 10 | 0 | 629 893 | 3 620 | 25 769 | 384 601 | 128 742 | 22 149 | 1 165 385 | 13 410 | 1 503 | 379 083 |  |
| 5 | 10 | 32 250 | 629 893 | 5 420 | 24 544 | 367 804 | 124 954 | 19 124 | 1 174 024 | 12 818 | 2 094 | 276 137 |  |

**e-Table 1 III: *R_0_*=2.5**

| **Target school** | **Duration (weeks)** | **Cost of lost learning ($1000)** | **Lost productivity due to school closure ($1000)** | **Energy savings ($1000)** | **Health care costs ($1000)** | **Lost productivity due to fatal cases ($1000)** | **Lost productivity due to sickness ($1000)** | **Cost of Oslo municipality ($1000)** | **Total cost ($1000)** | **QALY loss** | **QALY gain (relative)** | **ICER (compared to no intervention) ($ per QALY** | **ICER (relative)** |
| --- | --- | --- | --- | --- | --- | --- | --- | --- | --- | --- | --- | --- | --- |
| 0 | 0 | 0 | 0 | 0 | 32 961 | 479 607 | 155 079 | 32 961 | 667 646 | 17 056 |  |  |  |
| 3 | 1 | 3 225 | 0 | 180 | 32 928 | 479 185 | 155 005 | 32 748 | 670 162 | 17 040 | 16 | 160 991 |  |
| 3 | 3 | 9 675 | 0 | 540 | 32 544 | 474 295 | 154 205 | 32 004 | 670 179 | 16 861 | 195 | 88 |  |
| 3 | 4 | 12 900 | 0 | 720 | 32 367 | 472 045 | 153 864 | 31 647 | 670 456 | 16 779 | 277 | 1 000 | 3 380 |
| 3 | 2 | 6 450 | 0 | 360 | 32 801 | 477 565 | 154 728 | 32 441 | 671 184 | 16 981 | 75 | 9 676 | Dominated |
| 3 | 5 | 16 125 | 0 | 900 | 32 318 | 471 424 | 153 771 | 31 418 | 672 739 | 16 757 | 299 | 5 192 | 101 226 |
| 3 | 6 | 19 350 | 0 | 1 080 | 32 308 | 471 296 | 153 752 | 31 228 | 675 626 | 16 752 | 304 | 9 498 | 620 315 |
| 3 | 7 | 22 575 | 0 | 1 260 | 32 306 | 471 271 | 153 749 | 31 046 | 678 641 | 16 751 | 305 | 9 888 | 3 386 921 |
| 3 | 8 | 25 800 | 0 | 1 440 | 32 306 | 471 267 | 153 748 | 30 866 | 681 681 | 16 751 | 305 | 9 963 | 20 007 697 |
| 3 | 9 | 29 025 | 0 | 1 620 | 32 306 | 471 266 | 153 748 | 30 686 | 684 725 | 16 751 | 305 | 9 978 | 126 703 892 |
| 3 | 10 | 32 250 | 0 | 1 800 | 32 306 | 471 266 | 153 748 | 30 506 | 687 770 | 16 751 | 305 | 9 978 | 289 245 859 |
| 2 | 1 | 0 | 26 795 | 188 | 32 910 | 478 888 | 154 902 | 32 722 | 693 308 | 17 032 | 24 | 226 859 |  |
| 1 | 1 | 0 | 36 194 | 174 | 32 917 | 479 000 | 154 950 | 32 743 | 702 886 | 17 035 | 21 | 457 814 |  |
| 2 | 2 | 0 | 53 591 | 376 | 32 733 | 476 363 | 154 300 | 32 357 | 716 611 | 16 946 | 110 | 124 597 |  |
| 4 | 1 | 0 | 62 989 | 362 | 32 870 | 478 334 | 154 782 | 32 508 | 728 614 | 17 013 | 44 | 275 908 |  |
| 5 | 1 | 3 225 | 62 989 | 542 | 32 842 | 477 979 | 154 718 | 32 300 | 731 212 | 16 999 | 57 | 45 782 |  |
| 1 | 2 | 0 | 72 388 | 348 | 32 742 | 476 595 | 154 451 | 32 394 | 735 828 | 16 952 | 104 | 44 351 |  |
| 2 | 3 | 0 | 80 386 | 564 | 32 345 | 471 070 | 153 238 | 31 781 | 736 475 | 16 762 | 294 | 2 200 |  |
| 2 | 4 | 0 | 107 181 | 752 | 31 958 | 466 033 | 152 436 | 31 206 | 756 856 | 16 583 | 473 | 43 086 |  |
| 1 | 3 | 0 | 108 582 | 522 | 32 408 | 472 154 | 153 645 | 31 886 | 766 267 | 16 796 | 261 | 36 126 |  |
| 2 | 5 | 0 | 133 976 | 940 | 31 773 | 463 669 | 152 109 | 30 833 | 780 587 | 16 498 | 558 | 25 678 |  |
| 4 | 2 | 0 | 125 979 | 724 | 32 561 | 473 986 | 153 789 | 31 837 | 785 591 | 16 864 | 192 | 26 058 |  |
| 5 | 2 | 6 450 | 125 979 | 1 084 | 32 470 | 472 845 | 153 598 | 31 386 | 790 257 | 16 821 | 235 | 19 833 |  |
| 1 | 4 | 0 | 144 776 | 696 | 32 161 | 468 969 | 153 148 | 31 465 | 798 359 | 16 681 | 375 | 21 624 |  |
| 2 | 6 | 0 | 160 772 | 1 128 | 31 723 | 463 028 | 152 024 | 30 595 | 806 419 | 16 475 | 581 | 13 880 |  |
| 2 | 7 | 0 | 187 567 | 1 316 | 31 711 | 462 875 | 152 004 | 30 395 | 832 841 | 16 470 | 586 | 45 075 |  |
| 1 | 5 | 0 | 180 970 | 870 | 32 076 | 467 884 | 152 994 | 31 206 | 833 054 | 16 642 | 414 | 515 |  |
| 4 | 3 | 0 | 188 968 | 1 086 | 31 876 | 464 644 | 151 910 | 30 790 | 836 312 | 16 539 | 517 | 6 303 |  |
| 5 | 3 | 9 675 | 188 968 | 1 626 | 31 612 | 461 272 | 151 302 | 29 986 | 841 202 | 16 415 | 642 | 7 623 |  |
| 2 | 8 | 0 | 214 362 | 1 504 | 31 709 | 462 850 | 152 001 | 30 205 | 859 417 | 16 469 | 587 | 31 025 |  |
| 1 | 6 | 0 | 217 164 | 1 044 | 32 057 | 467 646 | 152 961 | 31 013 | 868 784 | 16 634 | 422 | 22 173 |  |
| 2 | 9 | 0 | 241 157 | 1 692 | 31 708 | 462 844 | 152 000 | 30 016 | 886 018 | 16 469 | 587 | 29 344 |  |
| 4 | 4 | 0 | 251 957 | 1 448 | 31 171 | 455 372 | 150 337 | 29 723 | 887 388 | 16 211 | 845 | 1 622 |  |
| 5 | 4 | 12 900 | 251 957 | 2 168 | 30 645 | 448 512 | 149 058 | 28 477 | 890 904 | 15 962 | 1 094 | 3 214 |  |
| 1 | 7 | 0 | 253 358 | 1 218 | 32 054 | 467 600 | 152 954 | 30 836 | 904 748 | 16 632 | 424 | 32 641 |  |
| 2 | 10 | 0 | 267 953 | 1 880 | 31 708 | 462 843 | 152 000 | 29 828 | 912 623 | 16 469 | 587 | 13 408 |  |
| 1 | 8 | 0 | 289 552 | 1 392 | 32 053 | 467 591 | 152 953 | 30 661 | 940 757 | 16 632 | 424 | 66 284 |  |
| 4 | 5 | 0 | 314 946 | 1 810 | 30 801 | 450 596 | 149 613 | 28 991 | 944 146 | 16 040 | 1 016 | 3 337 |  |
| 5 | 5 | 16 125 | 314 946 | 2 710 | 30 072 | 441 050 | 147 851 | 27 362 | 947 335 | 15 696 | 1 360 | 2 345 |  |
| 1 | 9 | 0 | 325 746 | 1 566 | 32 053 | 467 590 | 152 953 | 30 487 | 976 776 | 16 632 | 424 | 69 360 |  |
| 4 | 6 | 0 | 377 936 | 2 172 | 30 685 | 449 110 | 149 397 | 28 513 | 1 004 956 | 15 987 | 1 069 | 26 364 |  |
| 5 | 6 | 19 350 | 377 936 | 3 252 | 29 867 | 438 394 | 147 437 | 26 615 | 1 009 732 | 15 601 | 1 455 | 3 283 |  |
| 1 | 10 | 0 | 361 940 | 1 740 | 32 053 | 467 590 | 152 953 | 30 313 | 1 012 796 | 16 632 | 424 | 7 218 |  |
| 4 | 7 | 0 | 440 925 | 2 534 | 30 657 | 448 749 | 149 346 | 28 123 | 1 067 143 | 15 974 | 1 082 | 50 240 |  |
| 5 | 7 | 22 575 | 440 925 | 3 794 | 29 812 | 437 683 | 147 328 | 26 018 | 1 074 528 | 15 576 | 1 480 | 4 989 |  |
| 4 | 8 | 0 | 503 914 | 2 896 | 30 652 | 448 685 | 149 336 | 27 756 | 1 129 691 | 15 972 | 1 084 | 50 886 |  |
| 5 | 8 | 25 800 | 503 914 | 4 336 | 29 798 | 437 509 | 147 301 | 25 462 | 1 139 986 | 15 570 | 1 486 | 6 927 |  |
| 4 | 9 | 0 | 566 903 | 3 258 | 30 651 | 448 669 | 149 334 | 27 393 | 1 192 300 | 15 971 | 1 085 | 48 232 |  |
| 5 | 9 | 29 025 | 566 903 | 4 878 | 29 796 | 437 478 | 147 296 | 24 918 | 1 205 620 | 15 569 | 1 487 | 8 956 |  |
| 4 | 10 | 0 | 629 893 | 3 620 | 30 651 | 448 666 | 149 334 | 27 031 | 1 254 923 | 15 971 | 1 085 | 45 450 |  |
| 5 | 10 | 32 250 | 629 893 | 5 420 | 29 795 | 437 468 | 147 295 | 24 375 | 1 271 280 | 15 568 | 1 488 | 10 995 |  |

**e-Table 2 (Scenario C): Cost and health outcome according to type and duration of school closure (The most cost-effective option is highlighted)**

Note: the maximum willingness to pay is set to be NOK 500,000 or US$71,500 based on the government guidance ^28^

**e-Table 2 I: *R_0_***=1.5

| **Target school** | **Duration (weeks)** | **Cost of lost learning ($1000)** | **Lost productivity due to school closure ($1000)** | **Energy savings ($1000)** | **Health care costs ($1000)** | **Lost productivity due to fatal cases ($1000)** | **Lost productivity due to sickness ($1000)** | **Cost of Oslo municipality ($1000)** | **Total cost ($1000)** | **QALY loss** | **QALY gain (relative)** | **ICER (compared to no intervention) ($ per QALY** | **ICER (relative)** |
| --- | --- | --- | --- | --- | --- | --- | --- | --- | --- | --- | --- | --- | --- |
| 0 | 0 | 0 | 0 | 0 | 20 591 | 312 958 | 101 576 | 20 591 | 435 125 | 10 591 |  |  |  |
| 3 | 1 | 3 225 | 0 | 180 | 20 548 | 312 351 | 101 423 | 20 368 | 437 367 | 10 570 | 21 | 107 182 |  |
| 3 | 2 | 6 450 | 0 | 360 | 20 487 | 311 474 | 101 202 | 20 127 | 439 253 | 10 540 | 51 | 80 725 |  |
| 3 | 3 | 9 675 | 0 | 540 | 20 394 | 310 156 | 100 870 | 19 854 | 440 554 | 10 494 | 97 | 56 220 |  |
| 3 | 4 | 12 900 | 0 | 720 | 20 272 | 308 427 | 100 435 | 19 552 | 441 314 | 10 435 | 156 | 39 652 | 12 772 |
| 3 | 5 | 16 125 | 0 | 900 | 20 152 | 306 717 | 100 004 | 19 252 | 442 098 | 10 376 | 215 | 32 440 | 13 319 |
| 3 | 6 | 19 350 | 0 | 1 080 | 20 058 | 305 383 | 99 668 | 18 978 | 443 379 | 10 330 | 261 | 31 636 | 27 876 |
| 3 | 7 | 22 575 | 0 | 1 260 | 19 995 | 304 490 | 99 444 | 18 735 | 445 244 | 10 299 | 292 | 34 700 | 60 731 |
| 3 | 8 | 25 800 | 0 | 1 440 | 19 958 | 303 966 | 99 311 | 18 518 | 447 595 | 10 281 | 310 | 40 271 | 130 276 |
| 3 | 9 | 29 025 | 0 | 1 620 | 19 941 | 303 720 | 99 250 | 18 321 | 450 315 | 10 273 | 318 | 47 752 | 321 777 |
| 3 | 10 | 32 250 | 0 | 1 800 | 19 930 | 303 568 | 99 211 | 18 130 | 453 160 | 10 268 | 323 | 55 779 | 544 829 |
| 2 | 1 | 0 | 26 795 | 188 | 20 535 | 312 180 | 101 396 | 20 347 | 460 718 | 10 564 | 27 | 945 273 |  |
| 1 | 1 | 0 | 36 194 | 174 | 20 561 | 312 539 | 101 481 | 20 387 | 470 601 | 10 576 | 15 | 2 423 431 |  |
| 2 | 2 | 0 | 53 591 | 376 | 20 464 | 311 187 | 101 168 | 20 088 | 486 032 | 10 529 | 62 | 826 277 |  |
| 4 | 1 | 0 | 62 989 | 362 | 20 504 | 311 757 | 101 300 | 20 142 | 496 188 | 10 549 | 42 | 1 459 093 |  |
| 5 | 1 | 3 225 | 62 989 | 542 | 20 462 | 311 148 | 101 145 | 19 920 | 498 427 | 10 528 | 63 | 1 007 500 |  |
| 1 | 2 | 0 | 72 388 | 348 | 20 516 | 311 915 | 101 339 | 20 168 | 505 810 | 10 555 | 36 | 1 940 231 |  |
| 2 | 3 | 0 | 80 386 | 564 | 20 344 | 309 526 | 100 786 | 19 780 | 510 477 | 10 472 | 119 | 631 360 |  |
| 2 | 4 | 0 | 107 181 | 752 | 20 184 | 307 309 | 100 275 | 19 432 | 534 198 | 10 395 | 196 | 504 521 |  |
| 1 | 3 | 0 | 108 582 | 522 | 20 444 | 310 925 | 101 113 | 19 922 | 540 542 | 10 520 | 71 | 1 483 863 |  |
| 4 | 2 | 0 | 125 979 | 724 | 20 393 | 310 210 | 100 945 | 19 669 | 556 802 | 10 495 | 96 | 1 271 279 |  |
| 2 | 5 | 0 | 133 976 | 940 | 20 012 | 304 912 | 99 723 | 19 072 | 557 683 | 10 311 | 280 | 438 275 |  |
| 5 | 2 | 6 450 | 125 979 | 1 084 | 20 295 | 308 822 | 100 591 | 19 211 | 561 053 | 10 448 | 144 | 877 383 |  |
| 1 | 4 | 0 | 144 776 | 696 | 20 350 | 309 631 | 100 818 | 19 654 | 574 880 | 10 475 | 116 | 1 202 562 |  |
| 2 | 6 | 0 | 160 772 | 1 128 | 19 851 | 302 674 | 99 206 | 18 723 | 581 376 | 10 234 | 357 | 409 316 |  |
| 2 | 7 | 0 | 187 567 | 1 316 | 19 737 | 301 085 | 98 839 | 18 421 | 605 912 | 10 179 | 412 | 414 050 |  |
| 1 | 5 | 0 | 180 970 | 870 | 20 254 | 308 298 | 100 513 | 19 384 | 609 165 | 10 428 | 163 | 1 069 286 |  |
| 4 | 3 | 0 | 188 968 | 1 086 | 20 211 | 307 682 | 100 363 | 19 125 | 616 138 | 10 407 | 184 | 985 278 |  |
| 5 | 3 | 9 675 | 188 968 | 1 626 | 20 035 | 305 178 | 99 720 | 18 409 | 621 951 | 10 321 | 270 | 692 317 |  |
| 2 | 8 | 0 | 214 362 | 1 504 | 19 664 | 300 064 | 98 603 | 18 160 | 631 189 | 10 143 | 448 | 437 764 |  |
| 1 | 6 | 0 | 217 164 | 1 044 | 20 176 | 307 215 | 100 264 | 19 132 | 643 775 | 10 390 | 201 | 1 040 246 |  |
| 2 | 9 | 0 | 241 157 | 1 692 | 19 625 | 299 524 | 98 478 | 17 933 | 657 092 | 10 124 | 467 | 475 678 |  |
| 4 | 4 | 0 | 251 957 | 1 448 | 19 971 | 304 354 | 99 593 | 18 523 | 674 428 | 10 292 | 300 | 798 961 |  |
| 1 | 7 | 0 | 253 358 | 1 218 | 20 122 | 306 469 | 100 093 | 18 904 | 678 823 | 10 364 | 227 | 1 075 403 |  |
| 5 | 4 | 12 900 | 251 957 | 2 168 | 19 707 | 300 576 | 98 616 | 17 539 | 681 587 | 10 162 | 429 | 574 050 |  |
| 2 | 10 | 0 | 267 953 | 1 880 | 19 601 | 299 188 | 98 400 | 17 721 | 683 261 | 10 113 | 478 | 518 811 |  |
| 1 | 8 | 0 | 289 552 | 1 392 | 20 089 | 306 018 | 99 989 | 18 697 | 714 257 | 10 349 | 242 | 1 151 851 |  |
| 4 | 5 | 0 | 314 946 | 1 810 | 19 698 | 300 553 | 98 711 | 17 888 | 732 098 | 10 159 | 432 | 687 959 |  |
| 5 | 5 | 16 125 | 314 946 | 2 710 | 19 323 | 295 179 | 97 313 | 16 613 | 740 176 | 9 975 | 616 | 495 054 |  |
| 1 | 9 | 0 | 325 746 | 1 566 | 20 074 | 305 807 | 99 940 | 18 508 | 750 002 | 10 341 | 250 | 1 261 046 |  |
| 1 | 10 | 0 | 361 940 | 1 740 | 20 065 | 305 679 | 99 911 | 18 325 | 785 854 | 10 337 | 254 | 1 379 802 |  |
| 4 | 6 | 0 | 377 936 | 2 172 | 19 454 | 297 155 | 97 918 | 17 282 | 790 290 | 10 041 | 550 | 646 088 |  |
| 5 | 6 | 19 350 | 377 936 | 3 252 | 18 968 | 290 182 | 96 099 | 15 716 | 799 282 | 9 802 | 789 | 461 516 |  |
| 4 | 7 | 0 | 440 925 | 2 534 | 19 274 | 294 644 | 97 329 | 16 740 | 849 638 | 9 954 | 637 | 650 840 |  |
| 5 | 7 | 22 575 | 440 925 | 3 794 | 18 691 | 286 287 | 95 148 | 14 897 | 859 832 | 9 667 | 924 | 459 791 |  |
| 4 | 8 | 0 | 503 914 | 2 896 | 19 158 | 293 018 | 96 947 | 16 262 | 910 140 | 9 898 | 693 | 685 123 |  |
| 5 | 8 | 25 800 | 503 914 | 4 336 | 18 520 | 283 872 | 94 555 | 14 184 | 922 325 | 9 584 | 1 007 | 483 745 |  |
| 4 | 9 | 0 | 566 903 | 3 258 | 19 091 | 292 084 | 96 727 | 15 833 | 971 548 | 9 865 | 726 | 739 181 |  |
| 5 | 9 | 29 025 | 566 903 | 4 878 | 18 399 | 282 159 | 94 134 | 13 521 | 985 742 | 9 525 | 1 066 | 516 376 |  |
| 4 | 10 | 0 | 629 893 | 3 620 | 19 056 | 291 593 | 96 611 | 15 436 | 1 033 533 | 9 848 | 743 | 805 686 |  |
| 5 | 10 | 32 250 | 629 893 | 5 420 | 18 339 | 281 307 | 93 924 | 12 919 | 1 050 292 | 9 495 | 1 096 | 561 416 |  |

**e-Table 2 II: *R_0_***=2.0

| **Target school** | **Duration (weeks)** | **Cost of lost learning ($1000)** | **Lost productivity due to school closure ($1000)** | **Energy savings ($1000)** | **Health care costs ($1000)** | **Lost productivity due to fatal cases ($1000)** | **Lost productivity due to sickness ($1000)** | **Cost of Oslo municipality ($1000)** | **Total cost ($1000)** | **QALY loss** | **QALY gain (relative)** | **ICER (compared to no intervention) ($ per QALY** | **ICER (relative)** |
| --- | --- | --- | --- | --- | --- | --- | --- | --- | --- | --- | --- | --- | --- |
| 0 | 0 | 0 | 0 | 0 | 28 890 | 428 137 | 138 654 | 28 890 | 595 682 | 14 912 |  |  |  |
| 3 | 1 | 3 225 | 0 | 180 | 28 868 | 427 833 | 138 592 | 28 688 | 598 338 | 14 902 | 11 | 243 942 |  |
| 3 | 2 | 6 450 | 0 | 360 | 28 804 | 426 983 | 138 418 | 28 444 | 600 296 | 14 871 | 41 | 111 734 |  |
| 3 | 3 | 9 675 | 0 | 540 | 28 686 | 425 405 | 138 099 | 28 146 | 601 324 | 14 815 | 98 | 57 730 |  |
| 3 | 4 | 12 900 | 0 | 720 | 28 568 | 423 836 | 137 787 | 27 848 | 602 371 | 14 759 | 154 | 43 509 | 18 690 |
| 3 | 5 | 16 125 | 0 | 900 | 28 511 | 423 076 | 137 637 | 27 611 | 604 448 | 14 732 | 181 | 48 467 | 76 565 |
| 3 | 6 | 19 350 | 0 | 1 080 | 28 491 | 422 812 | 137 585 | 27 411 | 607 158 | 14 722 | 190 | 60 311 | 288 069 |
| 3 | 7 | 22 575 | 0 | 1 260 | 28 485 | 422 736 | 137 570 | 27 225 | 610 106 | 14 719 | 193 | 74 737 |  |
| 3 | 8 | 25 800 | 0 | 1 440 | 28 483 | 422 714 | 137 566 | 27 043 | 613 123 | 14 719 | 194 | 90 009 |  |
| 3 | 9 | 29 025 | 0 | 1 620 | 28 483 | 422 707 | 137 564 | 26 863 | 616 160 | 14 718 | 194 | 105 556 |  |
| 3 | 10 | 32 250 | 0 | 1 800 | 28 483 | 422 706 | 137 564 | 26 683 | 619 203 | 14 718 | 194 | 121 215 |  |
| 2 | 1 | 0 | 26 795 | 188 | 28 864 | 427 788 | 138 594 | 28 676 | 621 853 | 14 900 | 13 | 2 075 948 |  |
| 1 | 1 | 0 | 36 194 | 174 | 28 872 | 427 899 | 138 616 | 28 698 | 631 407 | 14 904 | 9 | 4 134 319 |  |
| 2 | 2 | 0 | 53 591 | 376 | 28 794 | 426 882 | 138 437 | 28 418 | 647 327 | 14 867 | 45 | 1 138 630 |  |
| 4 | 1 | 0 | 62 989 | 362 | 28 845 | 427 546 | 138 554 | 28 483 | 657 573 | 14 891 | 21 | 2 891 872 |  |
| 5 | 1 | 3 225 | 62 989 | 542 | 28 822 | 427 241 | 138 491 | 28 280 | 660 226 | 14 880 | 32 | 1 995 776 |  |
| 1 | 2 | 0 | 72 388 | 348 | 28 817 | 427 189 | 138 500 | 28 469 | 666 545 | 14 878 | 34 | 2 057 563 |  |
| 2 | 3 | 0 | 80 386 | 564 | 28 642 | 424 919 | 138 102 | 28 078 | 671 486 | 14 796 | 116 | 652 408 |  |
| 2 | 4 | 0 | 107 181 | 752 | 28 460 | 422 561 | 137 708 | 27 708 | 695 159 | 14 711 | 201 | 494 563 |  |
| 1 | 3 | 0 | 108 582 | 522 | 28 706 | 425 767 | 138 269 | 28 184 | 700 802 | 14 826 | 86 | 1 221 463 |  |
| 4 | 2 | 0 | 125 979 | 724 | 28 725 | 425 997 | 138 292 | 28 001 | 718 268 | 14 835 | 78 | 1 581 169 |  |
| 2 | 5 | 0 | 133 976 | 940 | 28 341 | 421 020 | 137 453 | 27 401 | 719 851 | 14 656 | 257 | 483 919 |  |
| 5 | 2 | 6 450 | 125 979 | 1 084 | 28 649 | 424 978 | 138 078 | 27 565 | 723 049 | 14 799 | 114 | 1 117 613 |  |
| 1 | 4 | 0 | 144 776 | 696 | 28 580 | 424 152 | 138 005 | 27 884 | 734 817 | 14 768 | 145 | 961 560 |  |
| 2 | 6 | 0 | 160 772 | 1 128 | 28 289 | 420 347 | 137 342 | 27 161 | 745 622 | 14 632 | 281 | 534 007 |  |
| 1 | 5 | 0 | 180 970 | 870 | 28 507 | 423 213 | 137 852 | 27 637 | 769 672 | 14 734 | 179 | 973 338 |  |
| 2 | 7 | 0 | 187 567 | 1 316 | 28 272 | 420 121 | 137 305 | 26 956 | 771 949 | 14 624 | 289 | 610 129 |  |
| 4 | 3 | 0 | 188 968 | 1 086 | 28 470 | 422 711 | 137 740 | 27 384 | 776 802 | 14 716 | 196 | 922 257 |  |
| 5 | 3 | 9 675 | 188 968 | 1 626 | 28 289 | 420 281 | 137 230 | 26 663 | 782 817 | 14 629 | 283 | 660 962 |  |
| 2 | 8 | 0 | 214 362 | 1 504 | 28 267 | 420 055 | 137 294 | 26 763 | 798 474 | 14 621 | 291 | 696 195 |  |
| 1 | 6 | 0 | 217 164 | 1 044 | 28 479 | 422 856 | 137 793 | 27 435 | 805 248 | 14 721 | 192 | 1 093 029 |  |
| 2 | 9 | 0 | 241 157 | 1 692 | 28 265 | 420 035 | 137 291 | 26 573 | 825 056 | 14 620 | 292 | 785 472 |  |
| 4 | 4 | 0 | 251 957 | 1 448 | 28 159 | 418 684 | 137 066 | 26 711 | 834 418 | 14 571 | 342 | 698 493 |  |
| 1 | 7 | 0 | 253 358 | 1 218 | 28 470 | 422 742 | 137 774 | 27 252 | 841 127 | 14 717 | 196 | 1 253 152 |  |
| 5 | 4 | 12 900 | 251 957 | 2 168 | 27 860 | 414 668 | 136 227 | 25 692 | 841 444 | 14 428 | 485 | 506 776 |  |
| 2 | 10 | 0 | 267 953 | 1 880 | 28 265 | 420 029 | 137 290 | 26 385 | 851 657 | 14 620 | 292 | 875 998 |  |
| 1 | 8 | 0 | 289 552 | 1 392 | 28 468 | 422 709 | 137 769 | 27 076 | 877 106 | 14 715 | 197 | 1 428 238 |  |
| 4 | 5 | 0 | 314 946 | 1 810 | 27 947 | 415 935 | 136 608 | 26 137 | 893 625 | 14 472 | 441 | 675 782 |  |
| 5 | 5 | 16 125 | 314 946 | 2 710 | 27 562 | 410 770 | 135 536 | 24 852 | 902 229 | 14 288 | 625 | 490 535 |  |
| 1 | 9 | 0 | 325 746 | 1 566 | 28 467 | 422 700 | 137 768 | 26 901 | 913 115 | 14 715 | 197 | 1 608 224 |  |
| 1 | 10 | 0 | 361 940 | 1 740 | 28 467 | 422 698 | 137 767 | 26 727 | 949 132 | 14 715 | 197 | 1 790 074 |  |
| 4 | 6 | 0 | 377 936 | 2 172 | 27 858 | 414 789 | 136 417 | 25 686 | 954 827 | 14 430 | 482 | 744 845 |  |
| 5 | 6 | 19 350 | 377 936 | 3 252 | 27 427 | 409 004 | 135 224 | 24 175 | 965 689 | 14 224 | 688 | 537 589 |  |
| 4 | 7 | 0 | 440 925 | 2 534 | 27 827 | 414 389 | 136 350 | 25 293 | 1 016 957 | 14 416 | 497 | 848 365 |  |
| 5 | 7 | 22 575 | 440 925 | 3 794 | 27 380 | 408 397 | 135 118 | 23 586 | 1 030 601 | 14 202 | 710 | 612 518 |  |
| 4 | 8 | 0 | 503 914 | 2 896 | 27 818 | 414 263 | 136 329 | 24 922 | 1 079 429 | 14 411 | 501 | 965 393 |  |
| 5 | 8 | 25 800 | 503 914 | 4 336 | 27 366 | 408 206 | 135 084 | 23 030 | 1 096 033 | 14 196 | 717 | 697 913 |  |
| 4 | 9 | 0 | 566 903 | 3 258 | 27 815 | 414 225 | 136 323 | 24 557 | 1 142 007 | 14 410 | 502 | 1 087 244 |  |
| 5 | 9 | 29 025 | 566 903 | 4 878 | 27 361 | 408 141 | 135 073 | 22 483 | 1 161 625 | 14 193 | 719 | 786 865 |  |
| 4 | 10 | 0 | 629 893 | 3 620 | 27 814 | 414 214 | 136 321 | 24 194 | 1 204 622 | 14 410 | 503 | 1 210 937 |  |
| 5 | 10 | 32 250 | 629 893 | 5 420 | 27 359 | 408 124 | 135 069 | 21 939 | 1 227 275 | 14 193 | 720 | 877 369 |  |

**e-Table 2 III: *R_0_***=2.5

| **Target school** | **Duration (weeks)** | **Cost of lost learning ($1000)** | **Lost productivity due to school closure ($1000)** | **Energy savings ($1000)** | **Health care costs ($1000)** | **Lost productivity due to fatal cases ($1000)** | **Lost productivity due to sickness ($1000)** | **Cost of Oslo municipality ($1000)** | **Total cost ($1000)** | **QALY loss** | **QALY gain (relative)** | **ICER (compared to no intervention) ($ per QALY** | **ICER (relative)** |
| --- | --- | --- | --- | --- | --- | --- | --- | --- | --- | --- | --- | --- | --- |
| 0 | 0 | 0 | 0 | 0 | 32 961 | 479 607 | 155 079 | 32 961 | 667 646 | 17 056 |  |  |  |
| 3 | 1 | 3 225 | 0 | 180 | 32 943 | 479 378 | 155 038 | 32 763 | 670 404 | 17 048 | 8 | 326 573 |  |
| 3 | 2 | 6 450 | 0 | 360 | 32 875 | 478 518 | 154 890 | 32 515 | 672 373 | 17 016 | 40 | 117 683 |  |
| 3 | 3 | 9 675 | 0 | 540 | 32 768 | 477 154 | 154 667 | 32 228 | 673 724 | 16 966 | 90 | 67 353 |  |
| 3 | 4 | 12 900 | 0 | 720 | 32 715 | 476 474 | 154 559 | 31 995 | 675 927 | 16 941 | 115 | 71 932 | 88 529 |
| 3 | 5 | 16 125 | 0 | 900 | 32 703 | 476 322 | 154 535 | 31 803 | 678 785 | 16 935 | 121 | 92 295 |  |
| 3 | 6 | 19 350 | 0 | 1 080 | 32 701 | 476 297 | 154 531 | 31 621 | 681 799 | 16 935 | 122 | 116 421 |  |
| 3 | 7 | 22 575 | 0 | 1 260 | 32 701 | 476 292 | 154 530 | 31 441 | 684 837 | 16 934 | 122 | 141 163 |  |
| 3 | 8 | 25 800 | 0 | 1 440 | 32 700 | 476 291 | 154 530 | 31 260 | 687 881 | 16 934 | 122 | 166 101 |  |
| 3 | 9 | 29 025 | 0 | 1 620 | 32 700 | 476 290 | 154 530 | 31 080 | 690 926 | 16 934 | 122 | 191 086 |  |
| 3 | 10 | 32 250 | 0 | 1 800 | 32 700 | 476 290 | 154 530 | 30 900 | 693 971 | 16 934 | 122 | 216 077 |  |
| 2 | 1 | 0 | 26 795 | 188 | 32 942 | 479 375 | 155 047 | 32 754 | 693 971 | 17 048 | 9 | 3 054 792 |  |
| 1 | 1 | 0 | 36 194 | 174 | 32 944 | 479 400 | 155 054 | 32 770 | 703 418 | 17 048 | 8 | 4 658 191 |  |
| 2 | 2 | 0 | 53 591 | 376 | 32 871 | 478 489 | 154 927 | 32 495 | 719 502 | 17 015 | 41 | 1 250 098 |  |
| 4 | 1 | 0 | 62 989 | 362 | 32 926 | 479 179 | 155 023 | 32 564 | 729 755 | 17 040 | 16 | 3 901 722 |  |
| 5 | 1 | 3 225 | 62 989 | 542 | 32 909 | 478 962 | 154 984 | 32 367 | 732 528 | 17 032 | 24 | 2 710 379 |  |
| 1 | 2 | 0 | 72 388 | 348 | 32 876 | 478 572 | 154 955 | 32 528 | 738 443 | 17 018 | 38 | 1 841 387 |  |
| 2 | 3 | 0 | 80 386 | 564 | 32 721 | 476 632 | 154 688 | 32 157 | 743 863 | 16 946 | 110 | 692 656 |  |
| 2 | 4 | 0 | 107 181 | 752 | 32 606 | 475 203 | 154 512 | 31 854 | 768 751 | 16 894 | 163 | 622 053 |  |
| 1 | 3 | 0 | 108 582 | 522 | 32 750 | 477 019 | 154 771 | 32 228 | 772 601 | 16 960 | 96 | 1 093 003 |  |
| 4 | 2 | 0 | 125 979 | 724 | 32 790 | 477 499 | 154 807 | 32 066 | 790 350 | 16 978 | 78 | 1 567 764 |  |
| 2 | 5 | 0 | 133 976 | 940 | 32 567 | 474 722 | 154 455 | 31 627 | 794 780 | 16 876 | 180 | 705 564 |  |
| 5 | 2 | 6 450 | 125 979 | 1 084 | 32 717 | 476 565 | 154 640 | 31 633 | 795 266 | 16 943 | 113 | 1 132 549 |  |
| 1 | 4 | 0 | 144 776 | 696 | 32 665 | 475 974 | 154 648 | 31 969 | 807 367 | 16 921 | 135 | 1 036 957 |  |
| 2 | 6 | 0 | 160 772 | 1 128 | 32 558 | 474 610 | 154 441 | 31 430 | 821 253 | 16 872 | 184 | 833 507 |  |
| 1 | 5 | 0 | 180 970 | 870 | 32 639 | 475 649 | 154 609 | 31 769 | 842 997 | 16 909 | 147 | 1 194 676 |  |
| 2 | 7 | 0 | 187 567 | 1 316 | 32 556 | 474 587 | 154 438 | 31 240 | 847 833 | 16 871 | 185 | 973 300 |  |
| 4 | 3 | 0 | 188 968 | 1 086 | 32 514 | 474 085 | 154 381 | 31 428 | 848 863 | 16 852 | 204 | 886 334 |  |
| 5 | 3 | 9 675 | 188 968 | 1 626 | 32 335 | 471 794 | 153 982 | 30 709 | 855 128 | 16 768 | 289 | 649 652 |  |
| 2 | 8 | 0 | 214 362 | 1 504 | 32 556 | 474 583 | 154 438 | 31 052 | 874 435 | 16 871 | 185 | 1 116 050 |  |
| 1 | 6 | 0 | 217 164 | 1 044 | 32 633 | 475 576 | 154 601 | 31 589 | 878 929 | 16 907 | 149 | 1 413 311 |  |
| 2 | 9 | 0 | 241 157 | 1 692 | 32 556 | 474 582 | 154 438 | 30 864 | 901 041 | 16 871 | 185 | 1 259 498 |  |
| 4 | 4 | 0 | 251 957 | 1 448 | 32 305 | 471 498 | 154 067 | 30 857 | 908 379 | 16 756 | 300 | 803 133 |  |
| 1 | 7 | 0 | 253 358 | 1 218 | 32 632 | 475 562 | 154 599 | 31 414 | 914 933 | 16 906 | 150 | 1 648 492 |  |
| 5 | 4 | 12 900 | 251 957 | 2 168 | 32 050 | 468 225 | 153 511 | 29 882 | 916 475 | 16 636 | 420 | 592 887 |  |
| 2 | 10 | 0 | 267 953 | 1 880 | 32 556 | 474 582 | 154 438 | 30 676 | 927 648 | 16 871 | 185 | 1 403 012 |  |
| 1 | 8 | 0 | 289 552 | 1 392 | 32 631 | 475 560 | 154 599 | 31 239 | 950 950 | 16 906 | 150 | 1 887 509 |  |
| 4 | 5 | 0 | 314 946 | 1 810 | 32 228 | 470 547 | 153 952 | 30 418 | 969 864 | 16 721 | 335 | 902 896 |  |
| 5 | 5 | 16 125 | 314 946 | 2 710 | 31 947 | 466 952 | 153 347 | 29 237 | 980 608 | 16 590 | 466 | 671 051 |  |
| 1 | 9 | 0 | 325 746 | 1 566 | 32 631 | 475 560 | 154 599 | 31 065 | 986 970 | 16 906 | 150 | 2 127 344 |  |
| 1 | 10 | 0 | 361 940 | 1 740 | 32 631 | 475 560 | 154 599 | 30 891 | 1 022 990 | 16 906 | 150 | 2 367 248 |  |
| 4 | 6 | 0 | 377 936 | 2 172 | 32 210 | 470 321 | 153 925 | 30 038 | 1 032 220 | 16 713 | 343 | 1 062 811 |  |
| 5 | 6 | 19 350 | 377 936 | 3 252 | 31 924 | 466 660 | 153 310 | 28 672 | 1 045 928 | 16 579 | 477 | 792 911 |  |
| 4 | 7 | 0 | 440 925 | 2 534 | 32 206 | 470 275 | 153 919 | 29 672 | 1 094 791 | 16 711 | 345 | 1 239 099 |  |
| 5 | 7 | 22 575 | 440 925 | 3 794 | 31 918 | 466 589 | 153 301 | 28 124 | 1 111 514 | 16 576 | 480 | 925 349 |  |
| 4 | 8 | 0 | 503 914 | 2 896 | 32 206 | 470 266 | 153 918 | 29 310 | 1 157 408 | 16 711 | 345 | 1 419 430 |  |
| 5 | 8 | 25 800 | 503 914 | 4 336 | 31 917 | 466 576 | 153 299 | 27 581 | 1 177 171 | 16 576 | 480 | 1 061 158 |  |
| 4 | 9 | 0 | 566 903 | 3 258 | 32 206 | 470 265 | 153 918 | 28 948 | 1 220 034 | 16 711 | 345 | 1 600 741 |  |
| 5 | 9 | 29 025 | 566 903 | 4 878 | 31 917 | 466 574 | 153 299 | 27 039 | 1 242 840 | 16 576 | 480 | 1 197 687 |  |
| 4 | 10 | 0 | 629 893 | 3 620 | 32 206 | 470 265 | 153 918 | 28 586 | 1 282 661 | 16 711 | 345 | 1 782 111 |  |
| 5 | 10 | 32 250 | 629 893 | 5 420 | 31 917 | 466 574 | 153 299 | 26 497 | 1 308 512 | 16 576 | 480 | 1 334 441 |  |

**e-Table 3 (Scenario D): Cost and health outcome according to type and duration of school closure (The most cost-effective option is highlighted)**

**e-Table 3 I: *R_0_*=1.5**

| **Target school** | **Duration (weeks)** | **Cost of lost learning ($1000)** | **Lost productivity due to school closure ($1000)** | **Energy savings ($1000)** | **Health care costs ($1000)** | **Lost productivity due to fatal cases ($1000)** | **Lost productivity due to sickness ($1000)** | **Cost of Oslo municipality ($1000)** | **Total cost ($1000)** | **QALY loss** | **QALY gain (relative)** | **ICER (compared to no intervention) ($ per QALY** | **ICER (relative)** |
| --- | --- | --- | --- | --- | --- | --- | --- | --- | --- | --- | --- | --- | --- |
| 0 | 0 | 0 | 0 | 0 | 20 591 | 3 129 580 | 101 576 | 20 591 | 3 251 748 | 76 011 | 0 |  |  |
| 3 | 9 | 29 025 | 0 | 1 620 | 19 264 | 2 940 636 | 96 784 | 17 644 | 3 084 090 | 71 297 | 4 715 | -35 562 |  |
| 3 | 8 | 25 800 | 0 | 1 440 | 19 318 | 2 948 253 | 96 978 | 17 878 | 3 088 909 | 71 487 | 4 525 | -35 989 | Dominated |
| 3 | 7 | 22 575 | 0 | 1 260 | 19 410 | 2 961 387 | 97 312 | 18 150 | 3 099 424 | 71 814 | 4 197 | -36 292 | Dominated |
| 3 | 6 | 19 350 | 0 | 1 080 | 19 557 | 2 982 392 | 97 846 | 18 477 | 3 118 065 | 72 338 | 3 673 | -36 393 | Dominated |
| 3 | 5 | 16 125 | 0 | 900 | 19 766 | 3 012 126 | 98 600 | 18 866 | 3 145 717 | 73 080 | 2 932 | -36 168 | Dominated |
| 3 | 4 | 12 900 | 0 | 720 | 20 008 | 3 046 614 | 99 474 | 19 288 | 3 178 276 | 73 940 | 2 071 | -35 476 | Dominated |
| 3 | 3 | 9 675 | 0 | 540 | 20 235 | 3 078 967 | 100 293 | 19 695 | 3 208 630 | 74 748 | 1 263 | -34 127 | Dominated |
| 2 | 7 | 0 | 187 567 | 1 316 | 19 117 | 2 924 186 | 96 793 | 17 801 | 3 226 347 | 70 840 | 5 172 | -4 912 | 311 207 |
| 3 | 2 | 6 450 | 0 | 360 | 20 403 | 3 102 869 | 100 897 | 20 043 | 3 230 259 | 75 345 | 667 | -32 229 | Dominated |
| 2 | 8 | 0 | 214 362 | 1 504 | 18 961 | 2 902 297 | 96 279 | 17 457 | 3 230 394 | 70 289 | 5 722 | -3 732 | 7 354 |
| 2 | 6 | 0 | 160 772 | 1 128 | 19 356 | 2 957 577 | 97 575 | 18 228 | 3 234 151 | 71 680 | 4 332 | -4 062 | Dominated |
| 2 | 9 | 0 | 241 157 | 1 692 | 18 858 | 2 887 995 | 95 942 | 17 166 | 3 242 261 | 69 930 | 6 082 | -1 560 | 33 001 |
| 3 | 1 | 3 225 | 0 | 180 | 20 509 | 3 117 923 | 101 278 | 20 329 | 3 242 756 | 75 721 | 291 | -30 916 | Dominated |
| 5 | 8 | 25 800 | 503 914 | 4 336 | 16 965 | 2 617 004 | 88 870 | 12 629 | 3 248 217 | 63 182 | 12 829 | -275 | 883 |
| 2 | 5 | 0 | 133 976 | 940 | 19 645 | 2 997 927 | 98 516 | 18 705 | 3 249 125 | 72 695 | 3 316 | -791 | Dominated |
| 5 | 7 | 22 575 | 440 925 | 3 794 | 17 426 | 2 682 833 | 90 555 | 13 632 | 3 250 519 | 64 824 | 11 188 | -110 | Dominated |
| 5 | 9 | 29 025 | 566 903 | 4 878 | 16 627 | 2 568 778 | 87 624 | 11 749 | 3 264 079 | 61 981 | 14 031 | 879 | 13 203 |
| 2 | 1 | 0 | 26 795 | 188 | 20 495 | 3 116 141 | 101 261 | 20 307 | 3 264 504 | 75 673 | 338 | 37 687 | Dominated |
| 2 | 4 | 0 | 107 181 | 752 | 19 950 | 3 040 392 | 99 504 | 19 198 | 3 266 275 | 73 764 | 2 247 | 6 465 | Dominated |
| 5 | 6 | 19 350 | 377 936 | 3 252 | 18 018 | 2 766 901 | 92 681 | 14 766 | 3 271 633 | 66 923 | 9 088 | 2 188 | Dominated |
| 2 | 2 | 0 | 53 591 | 376 | 20 385 | 3 100 944 | 100 909 | 20 009 | 3 275 452 | 75 290 | 721 | 32 857 | Dominated |
| 2 | 3 | 0 | 80 386 | 564 | 20 210 | 3 076 506 | 100 342 | 19 646 | 3 276 880 | 74 674 | 1 337 | 18 794 | Dominated |
| 1 | 1 | 0 | 36 194 | 174 | 20 530 | 3 121 205 | 101 383 | 20 356 | 3 279 138 | 75 800 | 212 | 129 498 |  |
| 5 | 1 | 3 225 | 62 989 | 542 | 20 367 | 3 098 171 | 100 816 | 19 825 | 3 285 026 | 75 223 | 789 | 42 195 | Dominated |
| 4 | 1 | 0 | 62 989 | 362 | 20 440 | 3 108 565 | 101 085 | 20 078 | 3 292 718 | 75 482 | 530 | 77 347 | Dominated |
| 5 | 5 | 16 125 | 314 946 | 2 710 | 18 630 | 2 853 627 | 94 847 | 15 920 | 3 295 465 | 69 092 | 6 920 | 6 318 | Dominated |
| 1 | 2 | 0 | 72 388 | 348 | 20 453 | 3 110 440 | 101 138 | 20 105 | 3 304 070 | 75 528 | 483 | 108 220 |  |
| 5 | 2 | 6 450 | 125 979 | 1 084 | 20 109 | 3 062 002 | 99 944 | 19 025 | 3 313 400 | 74 315 | 1 697 | 36 333 | Dominated |
| 5 | 4 | 12 900 | 251 957 | 2 168 | 19 239 | 2 939 595 | 96 967 | 17 071 | 3 318 490 | 71 244 | 4 767 | 14 001 | Dominated |
| 1 | 3 | 0 | 108 582 | 522 | 20 323 | 3 092 568 | 100 729 | 19 801 | 3 321 680 | 75 076 | 935 | 74 800 | Dominated |
| 5 | 3 | 9 675 | 188 968 | 1 626 | 19 736 | 3 009 516 | 98 673 | 18 110 | 3 324 942 | 72 997 | 3 014 | 24 285 | Dominated |
| 4 | 2 | 0 | 125 979 | 724 | 20 263 | 3 084 012 | 100 517 | 19 539 | 3 330 047 | 74 863 | 1 149 | 68 155 | Dominated |
| 1 | 4 | 0 | 144 776 | 696 | 20 148 | 3 068 357 | 100 173 | 19 452 | 3 332 758 | 74 465 | 1 546 | 52 392 | Dominated |
| 1 | 5 | 0 | 180 970 | 870 | 19 971 | 3 043 743 | 99 606 | 19 101 | 3 343 419 | 73 844 | 2 167 | 42 295 | Dominated |
| 4 | 3 | 0 | 188 968 | 1 086 | 19 989 | 3 045 898 | 99 632 | 18 903 | 3 353 401 | 73 902 | 2 110 | 48 188 | Dominated |
| 4 | 6 | 0 | 377 936 | 2 172 | 18 702 | 2 866 307 | 95 396 | 16 530 | 3 356 169 | 69 383 | 6 628 | 15 755 | Dominated |
| 1 | 6 | 0 | 217 164 | 1 044 | 19 812 | 3 021 700 | 99 095 | 18 768 | 3 356 727 | 73 288 | 2 723 | 38 546 | Dominated |
| 4 | 5 | 0 | 314 946 | 1 810 | 19 151 | 2 929 162 | 96 892 | 17 341 | 3 358 341 | 70 963 | 5 048 | 21 116 | Dominated |
| 4 | 4 | 0 | 251 957 | 1 448 | 19 608 | 2 992 901 | 98 394 | 18 160 | 3 361 411 | 72 567 | 3 444 | 31 839 | Dominated |
| 4 | 7 | 0 | 440 925 | 2 534 | 18 330 | 2 814 124 | 94 143 | 15 796 | 3 364 989 | 68 073 | 7 938 | 14 265 | Dominated |
| 1 | 7 | 0 | 253 358 | 1 218 | 19 703 | 3 006 539 | 98 742 | 18 485 | 3 377 124 | 72 906 | 3 106 | 40 369 | Dominated |
| 4 | 8 | 0 | 503 914 | 2 896 | 18 058 | 2 775 803 | 93 217 | 15 162 | 3 388 096 | 67 112 | 8 900 | 15 321 | Dominated |
| 1 | 8 | 0 | 289 552 | 1 392 | 19 639 | 2 997 667 | 98 536 | 18 247 | 3 404 002 | 72 682 | 3 329 | 45 730 | Dominated |
| 4 | 9 | 0 | 566 903 | 3 258 | 17 885 | 2 751 521 | 92 627 | 14 627 | 3 425 678 | 66 503 | 9 508 | 18 292 | Dominated |
| 1 | 9 | 0 | 325 746 | 1 566 | 19 605 | 2 993 017 | 98 427 | 18 039 | 3 435 230 | 72 565 | 3 447 | 53 235 | Dominated |

**e-Table 3 II: *R_0_***=2.0

| **Target school** | **Duration (weeks)** | **Cost of lost learning ($1000)** | **Lost productivity due to school closure ($1000)** | **Energy savings ($1000)** | **Health care costs ($1000)** | **Lost productivity due to fatal cases ($1000)** | **Lost productivity due to sickness ($1000)** | **Cost of Oslo municipality ($1000)** | **Total cost ($1000)** | **QALY loss** | **QALY gain (relative)** | **ICER (compared to no intervention) ($ per QALY** | **ICER (relative)** |
| --- | --- | --- | --- | --- | --- | --- | --- | --- | --- | --- | --- | --- | --- |
| 0 | 0 | 0 | 0 | 0 | 28 890 | 4 281 367 | 138 654 | 28 890 | 4 448 912 | 104 973 | 0 |  |  |
| 3 | 6 | 19 350 | 0 | 1 080 | 27 985 | 4 160 624 | 136 245 | 26 905 | 4 343 124 | 101 876 | 3 097 | -34 158 |  |
| 3 | 7 | 22 575 | 0 | 1 260 | 27 964 | 4 157 798 | 136 190 | 26 704 | 4 343 266 | 101 803 | 3 170 | -33 331 | 1 969 |
| 3 | 8 | 25 800 | 0 | 1 440 | 27 957 | 4 156 951 | 136 173 | 26 517 | 4 345 442 | 101 782 | 3 191 | -32 423 | 100 104 |
| 3 | 9 | 29 025 | 0 | 1 620 | 27 955 | 4 156 719 | 136 169 | 26 335 | 4 348 248 | 101 776 | 3 197 | -31 484 | 179 840 |
| 3 | 5 | 16 125 | 0 | 900 | 28 049 | 4 169 195 | 136 411 | 27 149 | 4 348 880 | 102 096 | 2 877 | -34 768 | Dominated |
| 3 | 4 | 12 900 | 0 | 720 | 28 215 | 4 191 353 | 136 843 | 27 495 | 4 368 592 | 102 665 | 2 308 | -34 794 | Dominated |
| 3 | 3 | 9 675 | 0 | 540 | 28 491 | 4 228 125 | 137 570 | 27 951 | 4 403 321 | 103 608 | 1 365 | -33 400 | Dominated |
| 5 | 5 | 16 125 | 314 946 | 2 710 | 26 272 | 3 937 578 | 132 340 | 23 562 | 4 424 552 | 96 096 | 8 877 | -2 744 | 13 435 |
| 5 | 6 | 19 350 | 377 936 | 3 252 | 25 798 | 3 875 021 | 131 192 | 22 546 | 4 426 045 | 94 484 | 10 489 | -2 180 | 926 |
| 3 | 2 | 6 450 | 0 | 360 | 28 732 | 4 260 179 | 138 216 | 28 372 | 4 433 217 | 104 430 | 543 | -28 909 | Dominated |
| 2 | 5 | 0 | 133 976 | 940 | 27 795 | 4 139 296 | 136 260 | 26 855 | 4 436 387 | 101 285 | 3 688 | -3 396 | Dominated |
| 2 | 6 | 0 | 160 772 | 1 128 | 27 636 | 4 118 693 | 135 925 | 26 508 | 4 441 898 | 100 750 | 4 223 | -1 661 | Dominated |
| 3 | 1 | 3 225 | 0 | 180 | 28 846 | 4 275 418 | 138 529 | 28 666 | 4 445 838 | 104 821 | 152 | -20 187 | Dominated |
| 2 | 4 | 0 | 107 181 | 752 | 28 108 | 4 179 815 | 136 924 | 27 356 | 4 451 275 | 102 337 | 2 636 | 896 | Dominated |
| 2 | 7 | 0 | 187 567 | 1 316 | 27 576 | 4 110 791 | 135 797 | 26 260 | 4 460 415 | 100 545 | 4 428 | 2 597 | Dominated |
| 5 | 4 | 12 900 | 251 957 | 2 168 | 27 050 | 4 039 975 | 134 214 | 24 882 | 4 463 928 | 98 738 | 6 235 | 2 408 | Dominated |
| 5 | 7 | 22 575 | 440 925 | 3 794 | 25 597 | 3 848 429 | 130 703 | 21 803 | 4 464 435 | 93 799 | 11 174 | 1 389 | 56 035 |
| 2 | 1 | 0 | 26 795 | 188 | 28 844 | 4 275 322 | 138 545 | 28 656 | 4 469 318 | 104 816 | 157 | 130 232 |  |
| 2 | 3 | 0 | 80 386 | 564 | 28 481 | 4 228 182 | 137 732 | 27 917 | 4 474 216 | 103 593 | 1 380 | 18 335 | Dominated |
| 1 | 1 | 0 | 36 194 | 174 | 28 853 | 4 276 571 | 138 575 | 28 679 | 4 480 019 | 104 848 | 125 | 248 901 |  |
| 2 | 2 | 0 | 53 591 | 376 | 28 735 | 4 261 122 | 138 297 | 28 359 | 4 481 368 | 104 448 | 525 | 61 808 | Dominated |
| 2 | 8 | 0 | 214 362 | 1 504 | 27 557 | 4 108 343 | 135 758 | 26 053 | 4 484 515 | 100 481 | 4 492 | 7 926 | Dominated |
| 5 | 1 | 3 225 | 62 989 | 542 | 28 769 | 4 265 464 | 138 354 | 28 227 | 4 498 259 | 104 562 | 411 | 120 103 |  |
| 4 | 1 | 0 | 62 989 | 362 | 28 810 | 4 270 961 | 138 472 | 28 448 | 4 500 870 | 104 703 | 270 | 192 204 |  |
| 1 | 2 | 0 | 72 388 | 348 | 28 752 | 4 263 620 | 138 363 | 28 404 | 4 502 775 | 104 510 | 463 | 116 420 |  |
| 1 | 3 | 0 | 108 582 | 522 | 28 535 | 4 235 761 | 137 909 | 28 013 | 4 510 265 | 103 784 | 1 189 | 51 606 | Dominated |
| 2 | 9 | 0 | 241 157 | 1 692 | 27 551 | 4 107 652 | 135 746 | 25 859 | 4 510 415 | 100 463 | 4 510 | 13 637 | Dominated |
| 1 | 4 | 0 | 144 776 | 696 | 28 275 | 4 202 306 | 137 363 | 27 579 | 4 512 024 | 102 912 | 2 061 | 30 628 | Dominated |
| 5 | 3 | 9 675 | 188 968 | 1 626 | 27 906 | 4 152 329 | 136 270 | 26 280 | 4 513 521 | 101 639 | 3 335 | 19 376 | Dominated |
| 4 | 5 | 0 | 314 946 | 1 810 | 27 034 | 4 040 838 | 134 582 | 25 224 | 4 515 591 | 98 727 | 6 246 | 10 675 | Dominated |
| 5 | 8 | 25 800 | 503 914 | 4 336 | 25 521 | 3 838 381 | 130 518 | 21 185 | 4 519 798 | 93 540 | 11 433 | 6 200 | 213 894 |
| 4 | 4 | 0 | 251 957 | 1 448 | 27 555 | 4 108 512 | 135 722 | 26 107 | 4 522 298 | 100 482 | 4 491 | 16 342 | Dominated |
| 1 | 5 | 0 | 180 970 | 870 | 28 108 | 4 180 786 | 137 011 | 27 238 | 4 526 005 | 102 352 | 2 621 | 29 412 | Dominated |
| 5 | 2 | 6 450 | 125 979 | 1 084 | 28 494 | 4 229 446 | 137 687 | 27 410 | 4 526 971 | 103 631 | 1 342 | 58 170 | Dominated |
| 4 | 2 | 0 | 125 979 | 724 | 28 618 | 4 246 076 | 138 047 | 27 894 | 4 537 996 | 104 056 | 917 | 97 123 | Dominated |
| 4 | 6 | 0 | 377 936 | 2 172 | 26 753 | 4 004 168 | 133 965 | 24 581 | 4 540 649 | 97 776 | 7 197 | 12 746 | Dominated |
| 4 | 3 | 0 | 188 968 | 1 086 | 28 179 | 4 189 299 | 137 085 | 27 093 | 4 542 444 | 102 580 | 2 393 | 39 091 | Dominated |
| 1 | 6 | 0 | 217 164 | 1 044 | 28 044 | 4 172 526 | 136 876 | 27 000 | 4 553 566 | 102 137 | 2 836 | 36 899 | Dominated |
| 5 | 9 | 29 025 | 566 903 | 4 878 | 25 494 | 3 834 889 | 130 454 | 20 616 | 4 581 888 | 93 450 | 11 523 | 11 540 | 690 371 |
| 1 | 7 | 0 | 253 358 | 1 218 | 28 022 | 4 169 755 | 136 830 | 26 804 | 4 586 748 | 102 065 | 2 908 | 47 393 | Dominated |
| 4 | 7 | 0 | 440 925 | 2 534 | 26 645 | 3 990 111 | 133 728 | 24 111 | 4 588 875 | 97 411 | 7 562 | 18 509 | Dominated |
| 1 | 8 | 0 | 289 552 | 1 392 | 28 017 | 4 169 018 | 136 818 | 26 625 | 4 622 013 | 102 045 | 2 928 | 59 128 | Dominated |
| 4 | 8 | 0 | 503 914 | 2 896 | 26 611 | 3 985 726 | 133 654 | 23 715 | 4 647 008 | 97 298 | 7 675 | 25 809 | Dominated |
| 1 | 9 | 0 | 325 746 | 1 566 | 28 015 | 4 168 814 | 136 815 | 26 449 | 4 657 825 | 102 040 | 2 933 | 71 232 | Dominated |
| 4 | 9 | 0 | 566 903 | 3 258 | 26 600 | 3 984 350 | 133 631 | 23 342 | 4 708 226 | 97 262 | 7 711 | 33 629 | Dominated |

**e-Table 3 III: *R_0_***=2.5

| **Target school** | **Duration (weeks)** | **Cost of lost learning ($1000)** | **Lost productivity due to school closure ($1000)** | **Energy savings ($1000)** | **Health care costs ($1000)** | **Lost productivity due to fatal cases ($1000)** | **Lost productivity due to sickness ($1000)** | **Cost of Oslo municipality ($1000)** | **Total cost ($1000)** | **QALY loss** | **QALY gain (relative)** | **ICER (compared to no intervention) ($ per QALY** | **ICER (relative)** |
| --- | --- | --- | --- | --- | --- | --- | --- | --- | --- | --- | --- | --- | --- |
| 0 | 0 | 0 | 0 | 0 | 32 961 | 4 796 068 | 155 079 | 32 961 | 4 984 107 | 118 410 | 0 |  |  |
| 3 | 5 | 16 125 | 0 | 900 | 32 318 | 4 714 238 | 153 771 | 31 418 | 4 915 553 | 116 269 | 2 140 | -32 030 |  |
| 3 | 6 | 19 350 | 0 | 1 080 | 32 308 | 4 712 956 | 153 752 | 31 228 | 4 917 287 | 116 236 | 2 174 | -30 738 |  |
| 3 | 4 | 12 900 | 0 | 720 | 32 367 | 4 720 447 | 153 864 | 31 647 | 4 918 858 | 116 432 | 1 978 | -32 992 | Dominated |
| 3 | 7 | 22 575 | 0 | 1 260 | 32 306 | 4 712 711 | 153 749 | 31 046 | 4 920 081 | 116 229 | 2 180 | -29 366 |  |
| 3 | 8 | 25 800 | 0 | 1 440 | 32 306 | 4 712 669 | 153 748 | 30 866 | 4 923 083 | 116 228 | 2 181 | -27 975 | 771 820 |
| 3 | 9 | 29 025 | 0 | 1 620 | 32 306 | 4 712 662 | 153 748 | 30 686 | 4 926 122 | 116 228 | 2 182 | -26 580 | 1 149 257 |
| 3 | 3 | 9 675 | 0 | 540 | 32 544 | 4 742 949 | 154 205 | 32 004 | 4 938 833 | 117 021 | 1 389 | -32 600 | Dominated |
| 3 | 2 | 6 450 | 0 | 360 | 32 801 | 4 775 655 | 154 728 | 32 441 | 4 969 274 | 117 876 | 533 | -27 817 | Dominated |
| 3 | 1 | 3 225 | 0 | 180 | 32 928 | 4 791 847 | 155 005 | 32 748 | 4 982 825 | 118 300 | 110 | -11 645 | Dominated |
| 2 | 4 | 0 | 107 181 | 752 | 32 174 | 4 698 352 | 153 856 | 31 422 | 4 990 812 | 115 824 | 2 586 | 2 593 | 178 482 |
| 5 | 4 | 12 900 | 251 957 | 2 168 | 30 971 | 4 546 626 | 151 625 | 28 803 | 4 991 911 | 111 837 | 6 573 | 1 187 | 16 964 |
| 2 | 5 | 0 | 133 976 | 940 | 32 013 | 4 678 357 | 153 631 | 31 073 | 4 997 036 | 115 295 | 3 115 | 4 150 | Dominated |
| 5 | 5 | 16 125 | 314 946 | 2 710 | 30 530 | 4 491 193 | 150 906 | 27 820 | 5 000 990 | 110 377 | 8 033 | 2 102 | 6 219 |
| 2 | 3 | 0 | 80 386 | 564 | 32 504 | 4 739 324 | 154 337 | 31 940 | 5 005 987 | 116 908 | 1 501 | 14 574 | Dominated |
| 2 | 1 | 0 | 26 795 | 188 | 32 929 | 4 792 103 | 155 022 | 32 741 | 5 006 661 | 118 305 | 105 | 215 188 |  |
| 1 | 1 | 0 | 36 194 | 174 | 32 929 | 4 792 159 | 155 031 | 32 755 | 5 016 138 | 118 306 | 104 | 308 219 |  |
| 2 | 6 | 0 | 160 772 | 1 128 | 31 970 | 4 673 020 | 153 571 | 30 842 | 5 018 205 | 115 153 | 3 256 | 10 471 | Dominated |
| 2 | 2 | 0 | 53 591 | 376 | 32 811 | 4 777 496 | 154 823 | 32 435 | 5 018 344 | 117 919 | 491 | 69 706 | Dominated |
| 5 | 3 | 9 675 | 188 968 | 1 626 | 31 806 | 4 651 428 | 153 021 | 30 180 | 5 033 272 | 114 598 | 3 812 | 12 897 | Dominated |
| 1 | 2 | 0 | 72 388 | 348 | 32 797 | 4 776 000 | 154 839 | 32 449 | 5 035 677 | 117 876 | 534 | 96 659 |  |
| 5 | 1 | 3 225 | 62 989 | 542 | 32 871 | 4 784 841 | 154 908 | 32 329 | 5 038 293 | 118 114 | 296 | 183 131 |  |
| 1 | 3 | 0 | 108 582 | 522 | 32 532 | 4 743 317 | 154 456 | 32 010 | 5 038 365 | 117 008 | 1 402 | 38 695 | Dominated |
| 4 | 1 | 0 | 62 989 | 362 | 32 899 | 4 788 441 | 154 977 | 32 537 | 5 038 944 | 118 208 | 202 | 271 264 |  |
| 2 | 7 | 0 | 187 567 | 1 316 | 31 962 | 4 672 001 | 153 560 | 30 646 | 5 043 773 | 115 126 | 3 283 | 18 172 | Dominated |
| 1 | 4 | 0 | 144 776 | 696 | 32 315 | 4 716 630 | 154 148 | 31 619 | 5 047 173 | 116 298 | 2 111 | 29 872 | Dominated |
| 5 | 6 | 19 350 | 377 936 | 3 252 | 30 395 | 4 474 246 | 150 689 | 27 143 | 5 049 363 | 109 931 | 8 479 | 7 696 | 108 396 |
| 4 | 4 | 0 | 251 957 | 1 448 | 31 531 | 4 618 951 | 152 891 | 30 083 | 5 053 883 | 113 718 | 4 692 | 14 872 | Dominated |
| 5 | 2 | 6 450 | 125 979 | 1 084 | 32 558 | 4 745 717 | 154 337 | 31 474 | 5 063 957 | 117 083 | 1 327 | 60 165 | Dominated |
| 4 | 3 | 0 | 188 968 | 1 086 | 32 117 | 4 691 649 | 153 760 | 31 031 | 5 065 408 | 115 643 | 2 767 | 29 381 | Dominated |
| 2 | 8 | 0 | 214 362 | 1 504 | 31 960 | 4 671 812 | 153 558 | 30 456 | 5 070 188 | 115 121 | 3 288 | 26 177 | Dominated |
| 4 | 2 | 0 | 125 979 | 724 | 32 671 | 4 760 221 | 154 614 | 31 947 | 5 072 760 | 117 459 | 950 | 93 293 |  |
| 1 | 5 | 0 | 180 970 | 870 | 32 242 | 4 707 712 | 154 046 | 31 372 | 5 074 101 | 116 062 | 2 348 | 38 326 | Dominated |
| 4 | 5 | 0 | 314 946 | 1 810 | 31 242 | 4 582 968 | 152 470 | 29 432 | 5 079 816 | 112 766 | 5 644 | 16 958 | Dominated |
| 2 | 9 | 0 | 241 157 | 1 692 | 31 960 | 4 671 768 | 153 557 | 30 268 | 5 096 750 | 115 120 | 3 290 | 34 242 | Dominated |
| 1 | 6 | 0 | 217 164 | 1 044 | 32 226 | 4 705 729 | 154 023 | 31 182 | 5 108 099 | 116 009 | 2 401 | 51 646 | Dominated |
| 5 | 7 | 22 575 | 440 925 | 3 794 | 30 360 | 4 469 901 | 150 633 | 26 566 | 5 110 600 | 109 816 | 8 593 | 14 720 | 195 494 |
| 4 | 6 | 0 | 377 936 | 2 172 | 31 161 | 4 572 899 | 152 353 | 28 989 | 5 132 177 | 112 499 | 5 910 | 25 053 | Dominated |
| 1 | 7 | 0 | 253 358 | 1 218 | 32 224 | 4 705 385 | 154 019 | 31 006 | 5 143 768 | 116 000 | 2 410 | 66 252 | Dominated |
| 5 | 8 | 25 800 | 503 914 | 4 336 | 30 354 | 4 469 104 | 150 623 | 26 018 | 5 175 459 | 109 795 | 8 614 | 22 213 | 299 951 |
| 1 | 8 | 0 | 289 552 | 1 392 | 32 223 | 4 705 321 | 154 019 | 30 831 | 5 179 723 | 115 998 | 2 412 | 81 114 |  |
| 4 | 7 | 0 | 440 925 | 2 534 | 31 145 | 4 570 919 | 152 330 | 28 611 | 5 192 784 | 112 447 | 5 963 | 34 998 | Dominated |
| 1 | 9 | 0 | 325 746 | 1 566 | 32 223 | 4 705 311 | 154 019 | 30 657 | 5 215 733 | 115 998 | 2 412 | 96 035 |  |
| 5 | 9 | 29 025 | 566 903 | 4 878 | 30 353 | 4 468 929 | 150 621 | 25 475 | 5 240 953 | 109 791 | 8 619 | 29 800 | 409 315 |
| 4 | 8 | 0 | 503 914 | 2 896 | 31 142 | 4 570 546 | 152 326 | 28 246 | 5 255 032 | 112 437 | 5 972 | 45 362 | Dominated |
| 4 | 9 | 0 | 566 903 | 3 258 | 31 142 | 4 570 467 | 152 325 | 27 884 | 5 317 578 | 112 435 | 5 975 | 55 815 | Dominated |

**e-Table 4 (Scenario E): Cost and health outcome according to type and duration of school closure (The most cost-effective option is highlighted)**

**e-Table 4 I: *R_0_***=1.5

| **Target school** | **Duration (weeks)** | **Cost of lost learning ($1000)** | **Lost productivity due to school closure ($1000)** | **Energy savings ($1000)** | **Health care costs ($1000)** | **Lost productivity due to fatal cases ($1000)** | **Lost productivity due to sickness ($1000)** | **Cost of Oslo municipality ($1000)** | **Total cost ($1000)** | **QALY loss** | **QALY gain (relative)** | **ICER (compared to no intervention) ($ per QALY** | **ICER (relative)** |
| --- | --- | --- | --- | --- | --- | --- | --- | --- | --- | --- | --- | --- | --- |
| 0 | 0 | 0 | 0 | 0 | 20 591 | 31 296 | 101 576 | 20 591 | 153 463 | 4 049 | 0 |  |  |
| 3 | 1 | 3 225 | 0 | 180 | 20 509 | 31 179 | 101 278 | 20 329 | 156 012 | 4 034 | 15 | 168 654 |  |
| 3 | 2 | 6 450 | 0 | 360 | 20 403 | 31 029 | 100 897 | 20 043 | 158 419 | 4 014 | 35 | 143 343 |  |
| 3 | 3 | 9 675 | 0 | 540 | 20 235 | 30 790 | 100 293 | 19 695 | 160 453 | 3 984 | 65 | 106 789 |  |
| 3 | 4 | 12 900 | 0 | 720 | 20 008 | 30 466 | 99 474 | 19 288 | 162 128 | 3 942 | 107 | 80 832 |  |
| 3 | 5 | 16 125 | 0 | 900 | 19 766 | 30 121 | 98 600 | 18 866 | 163 712 | 3 897 | 152 | 67 588 |  |
| 3 | 6 | 19 350 | 0 | 1 080 | 19 557 | 29 824 | 97 846 | 18 477 | 165 497 | 3 859 | 190 | 63 362 | 46 621 |
| 3 | 7 | 22 575 | 0 | 1 260 | 19 410 | 29 614 | 97 312 | 18 150 | 167 651 | 3 832 | 217 | 65 399 | 79 714 |
| 3 | 8 | 25 800 | 0 | 1 440 | 19 318 | 29 483 | 96 978 | 17 878 | 170 138 | 3 815 | 234 | 71 314 | 147 306 |
| 3 | 9 | 29 025 | 0 | 1 620 | 19 264 | 29 406 | 96 784 | 17 644 | 172 859 | 3 805 | 244 | 79 619 |  |
| 2 | 1 | 0 | 26 795 | 188 | 20 495 | 31 161 | 101 261 | 20 307 | 179 524 | 4 031 | 18 | 1 481 710 |  |
| 1 | 1 | 0 | 36 194 | 174 | 20 530 | 31 212 | 101 383 | 20 356 | 189 146 | 4 038 | 11 | 3 228 281 |  |
| 2 | 2 | 0 | 53 591 | 376 | 20 385 | 31 009 | 100 909 | 20 009 | 205 518 | 4 012 | 37 | 1 391 099 |  |
| 4 | 1 | 0 | 62 989 | 362 | 20 440 | 31 086 | 101 085 | 20 078 | 215 238 | 4 021 | 28 | 2 238 737 |  |
| 5 | 1 | 3 225 | 62 989 | 542 | 20 367 | 30 982 | 100 816 | 19 825 | 217 837 | 4 008 | 41 | 1 567 609 |  |
| 1 | 2 | 0 | 72 388 | 348 | 20 453 | 31 104 | 101 138 | 20 105 | 224 735 | 4 024 | 25 | 2 825 567 |  |
| 2 | 3 | 0 | 80 386 | 564 | 20 210 | 30 765 | 100 342 | 19 646 | 231 139 | 3 980 | 69 | 1 121 249 |  |
| 2 | 4 | 0 | 107 181 | 752 | 19 950 | 30 404 | 99 504 | 19 198 | 256 287 | 3 933 | 116 | 884 210 |  |
| 1 | 3 | 0 | 108 582 | 522 | 20 323 | 30 926 | 100 729 | 19 801 | 260 038 | 4 000 | 49 | 2 186 545 |  |
| 4 | 2 | 0 | 125 979 | 724 | 20 263 | 30 840 | 100 517 | 19 539 | 276 875 | 3 989 | 60 | 2 065 591 |  |
| 2 | 5 | 0 | 133 976 | 940 | 19 645 | 29 979 | 98 516 | 18 705 | 281 177 | 3 878 | 171 | 744 767 |  |
| 5 | 2 | 6 450 | 125 979 | 1 084 | 20 109 | 30 620 | 99 944 | 19 025 | 282 018 | 3 961 | 88 | 1 457 417 |  |
| 1 | 4 | 0 | 144 776 | 696 | 20 148 | 30 684 | 100 173 | 19 452 | 295 085 | 3 968 | 81 | 1 757 451 |  |
| 2 | 6 | 0 | 160 772 | 1 128 | 19 356 | 29 576 | 97 575 | 18 228 | 306 150 | 3 825 | 224 | 682 099 |  |
| 1 | 5 | 0 | 180 970 | 870 | 19 971 | 30 437 | 99 606 | 19 101 | 330 114 | 3 936 | 113 | 1 564 140 |  |
| 2 | 7 | 0 | 187 567 | 1 316 | 19 117 | 29 242 | 96 793 | 17 801 | 331 402 | 3 782 | 267 | 666 111 |  |
| 4 | 3 | 0 | 188 968 | 1 086 | 19 989 | 30 459 | 99 632 | 18 903 | 337 962 | 3 939 | 110 | 1 683 387 |  |
| 5 | 3 | 9 675 | 188 968 | 1 626 | 19 736 | 30 095 | 98 673 | 18 110 | 345 521 | 3 892 | 157 | 1 226 962 |  |
| 2 | 8 | 0 | 214 362 | 1 504 | 18 961 | 29 023 | 96 279 | 17 457 | 357 120 | 3 754 | 295 | 689 239 |  |
| 1 | 6 | 0 | 217 164 | 1 044 | 19 812 | 30 217 | 99 095 | 18 768 | 365 244 | 3 907 | 142 | 1 492 484 |  |
| 2 | 9 | 0 | 241 157 | 1 692 | 18 858 | 28 880 | 95 942 | 17 166 | 383 146 | 3 735 | 314 | 731 483 |  |
| 4 | 4 | 0 | 251 957 | 1 448 | 19 608 | 29 929 | 98 394 | 18 160 | 398 440 | 3 870 | 179 | 1 369 939 |  |
| 1 | 7 | 0 | 253 358 | 1 218 | 19 703 | 30 065 | 98 742 | 18 485 | 400 651 | 3 887 | 162 | 1 527 659 |  |
| 5 | 4 | 12 900 | 251 957 | 2 168 | 19 239 | 29 396 | 96 967 | 17 071 | 408 291 | 3 802 | 247 | 1 029 941 |  |
| 1 | 8 | 0 | 289 552 | 1 392 | 19 639 | 29 977 | 98 536 | 18 247 | 436 311 | 3 876 | 173 | 1 630 654 |  |
| 4 | 5 | 0 | 314 946 | 1 810 | 19 151 | 29 292 | 96 892 | 17 341 | 458 471 | 3 787 | 262 | 1 164 456 |  |
| 5 | 5 | 16 125 | 314 946 | 2 710 | 18 630 | 28 536 | 94 847 | 15 920 | 470 374 | 3 690 | 359 | 882 804 |  |
| 1 | 9 | 0 | 325 746 | 1 566 | 19 605 | 29 930 | 98 427 | 18 039 | 472 143 | 3 869 | 180 | 1 774 769 |  |
| 4 | 6 | 0 | 377 936 | 2 172 | 18 702 | 28 663 | 95 396 | 16 530 | 518 525 | 3 705 | 344 | 1 062 010 |  |
| 5 | 6 | 19 350 | 377 936 | 3 252 | 18 018 | 27 669 | 92 681 | 14 766 | 532 401 | 3 578 | 471 | 803 949 |  |
| 4 | 7 | 0 | 440 925 | 2 534 | 18 330 | 28 141 | 94 143 | 15 796 | 579 006 | 3 637 | 412 | 1 033 951 |  |
| 5 | 7 | 22 575 | 440 925 | 3 794 | 17 426 | 26 828 | 90 555 | 13 632 | 594 515 | 3 469 | 580 | 760 285 |  |
| 4 | 8 | 0 | 503 914 | 2 896 | 18 058 | 27 758 | 93 217 | 15 162 | 640 051 | 3 588 | 461 | 1 054 769 |  |
| 5 | 8 | 25 800 | 503 914 | 4 336 | 16 965 | 26 170 | 88 870 | 12 629 | 657 383 | 3 384 | 665 | 757 555 |  |
| 4 | 9 | 0 | 566 903 | 3 258 | 17 885 | 27 515 | 92 627 | 14 627 | 701 672 | 3 556 | 493 | 1 112 383 |  |
| 5 | 9 | 29 025 | 566 903 | 4 878 | 16 627 | 25 688 | 87 624 | 11 749 | 720 989 | 3 322 | 727 | 780 136 |  |

**e-Table 4 II: *R_0_***=2.0

| **Target school** | **Duration (weeks)** | **Cost of lost learning ($1000)** | **Lost productivity due to school closure ($1000)** | **Energy savings ($1000)** | **Health care costs ($1000)** | **Lost productivity due to fatal cases ($1000)** | **Lost productivity due to sickness ($1000)** | **Cost of Oslo municipality ($1000)** | **Total cost ($1000)** | **QALY loss** | **QALY gain (relative)** | **ICER (compared to no intervention) ($ per QALY** | **ICER (relative)** |
| --- | --- | --- | --- | --- | --- | --- | --- | --- | --- | --- | --- | --- | --- |
| 0 | 0 | 0 | 0 | 0 | 28 890 | 42 814 | 138 654 | 28 890 | 210 359 | 5 906 | 0 |  |  |
| 3 | 1 | 3 225 | 0 | 180 | 28 846 | 42 754 | 138 529 | 28 666 | 213 174 | 5 898 | 8 | 341 664 |  |
| 3 | 2 | 6 450 | 0 | 360 | 28 732 | 42 602 | 138 216 | 28 372 | 215 640 | 5 877 | 29 | 181 029 |  |
| 3 | 3 | 9 675 | 0 | 540 | 28 491 | 42 281 | 137 570 | 27 951 | 217 477 | 5 834 | 73 | 97 623 |  |
| 3 | 4 | 12 900 | 0 | 720 | 28 215 | 41 914 | 136 843 | 27 495 | 219 152 | 5 784 | 123 | 71 646 |  |
| 3 | 5 | 16 125 | 0 | 900 | 28 049 | 41 692 | 136 411 | 27 149 | 221 377 | 5 754 | 153 | 72 200 |  |
| 3 | 6 | 19 350 | 0 | 1 080 | 27 985 | 41 606 | 136 245 | 26 905 | 224 106 | 5 742 | 164 | 83 753 |  |
| 3 | 7 | 22 575 | 0 | 1 260 | 27 964 | 41 578 | 136 190 | 26 704 | 227 046 | 5 738 | 168 | 99 370 |  |
| 3 | 8 | 25 800 | 0 | 1 440 | 27 957 | 41 570 | 136 173 | 26 517 | 230 060 | 5 737 | 169 | 116 526 |  |
| 3 | 9 | 29 025 | 0 | 1 620 | 27 955 | 41 567 | 136 169 | 26 335 | 233 096 | 5 737 | 169 | 134 236 |  |
| 2 | 1 | 0 | 26 795 | 188 | 28 844 | 42 753 | 138 545 | 28 656 | 236 749 | 5 898 | 8 | 3 128 018 |  |
| 1 | 1 | 0 | 36 194 | 174 | 28 853 | 42 766 | 138 575 | 28 679 | 246 213 | 5 900 | 7 | 5 350 329 |  |
| 2 | 2 | 0 | 53 591 | 376 | 28 735 | 42 611 | 138 297 | 28 359 | 262 857 | 5 878 | 28 | 1 870 125 |  |
| 4 | 1 | 0 | 62 989 | 362 | 28 810 | 42 710 | 138 472 | 28 448 | 272 619 | 5 892 | 15 | 4 281 664 |  |
| 5 | 1 | 3 225 | 62 989 | 542 | 28 769 | 42 655 | 138 354 | 28 227 | 275 450 | 5 884 | 22 | 2 936 296 |  |
| 1 | 2 | 0 | 72 388 | 348 | 28 752 | 42 636 | 138 363 | 28 404 | 281 792 | 5 882 | 25 | 2 893 306 |  |
| 2 | 3 | 0 | 80 386 | 564 | 28 481 | 42 282 | 137 732 | 27 917 | 288 316 | 5 833 | 73 | 1 063 295 |  |
| 2 | 4 | 0 | 107 181 | 752 | 28 108 | 41 798 | 136 924 | 27 356 | 313 259 | 5 767 | 139 | 739 436 |  |
| 1 | 3 | 0 | 108 582 | 522 | 28 535 | 42 358 | 137 909 | 28 013 | 316 862 | 5 843 | 63 | 1 682 451 |  |
| 4 | 2 | 0 | 125 979 | 724 | 28 618 | 42 461 | 138 047 | 27 894 | 334 380 | 5 857 | 49 | 2 529 427 |  |
| 2 | 5 | 0 | 133 976 | 940 | 27 795 | 41 393 | 136 260 | 26 855 | 338 484 | 5 713 | 194 | 660 776 |  |
| 5 | 2 | 6 450 | 125 979 | 1 084 | 28 494 | 42 294 | 137 687 | 27 410 | 339 820 | 5 834 | 72 | 1 799 245 |  |
| 1 | 4 | 0 | 144 776 | 696 | 28 275 | 42 023 | 137 363 | 27 579 | 351 741 | 5 797 | 110 | 1 290 606 |  |
| 2 | 6 | 0 | 160 772 | 1 128 | 27 636 | 41 187 | 135 925 | 26 508 | 364 392 | 5 685 | 222 | 695 031 |  |
| 1 | 5 | 0 | 180 970 | 870 | 28 108 | 41 808 | 137 011 | 27 238 | 387 027 | 5 767 | 139 | 1 268 882 |  |
| 2 | 7 | 0 | 187 567 | 1 316 | 27 576 | 41 108 | 135 797 | 26 260 | 390 731 | 5 674 | 232 | 776 685 |  |
| 4 | 3 | 0 | 188 968 | 1 086 | 28 179 | 41 893 | 137 085 | 27 093 | 395 038 | 5 779 | 127 | 1 450 600 |  |
| 5 | 3 | 9 675 | 188 968 | 1 626 | 27 906 | 41 523 | 136 270 | 26 280 | 402 715 | 5 729 | 178 | 1 081 141 |  |
| 2 | 8 | 0 | 214 362 | 1 504 | 27 557 | 41 083 | 135 758 | 26 053 | 417 256 | 5 671 | 236 | 878 466 |  |
| 1 | 6 | 0 | 217 164 | 1 044 | 28 044 | 41 725 | 136 876 | 27 000 | 422 765 | 5 756 | 151 | 1 410 262 |  |
| 2 | 9 | 0 | 241 157 | 1 692 | 27 551 | 41 077 | 135 746 | 25 859 | 443 840 | 5 670 | 236 | 987 450 |  |
| 4 | 4 | 0 | 251 957 | 1 448 | 27 555 | 41 085 | 135 722 | 26 107 | 454 871 | 5 669 | 238 | 1 027 930 |  |
| 1 | 7 | 0 | 253 358 | 1 218 | 28 022 | 41 698 | 136 830 | 26 804 | 458 690 | 5 752 | 154 | 1 608 028 |  |
| 5 | 4 | 12 900 | 251 957 | 2 168 | 27 050 | 40 400 | 134 214 | 24 882 | 464 353 | 5 575 | 331 | 767 076 |  |
| 1 | 8 | 0 | 289 552 | 1 392 | 28 017 | 41 690 | 136 818 | 26 625 | 494 685 | 5 751 | 155 | 1 829 063 |  |
| 4 | 5 | 0 | 314 946 | 1 810 | 27 034 | 40 408 | 134 582 | 25 224 | 515 162 | 5 577 | 330 | 924 113 |  |
| 5 | 5 | 16 125 | 314 946 | 2 710 | 26 272 | 39 376 | 132 340 | 23 562 | 526 349 | 5 437 | 470 | 672 635 |  |
| 1 | 9 | 0 | 325 746 | 1 566 | 28 015 | 41 688 | 136 815 | 26 449 | 530 698 | 5 751 | 156 | 2 057 032 |  |
| 4 | 6 | 0 | 377 936 | 2 172 | 26 753 | 40 042 | 133 965 | 24 581 | 576 523 | 5 527 | 379 | 964 993 |  |
| 5 | 6 | 19 350 | 377 936 | 3 252 | 25 798 | 38 750 | 131 192 | 22 546 | 589 774 | 5 352 | 554 | 684 721 |  |
| 4 | 7 | 0 | 440 925 | 2 534 | 26 645 | 39 901 | 133 728 | 24 111 | 638 664 | 5 508 | 398 | 1 074 989 |  |
| 5 | 7 | 22 575 | 440 925 | 3 794 | 25 597 | 38 484 | 130 703 | 21 803 | 654 490 | 5 317 | 590 | 752 907 |  |
| 4 | 8 | 0 | 503 914 | 2 896 | 26 611 | 39 857 | 133 654 | 23 715 | 701 140 | 5 502 | 404 | 1 213 765 |  |
| 5 | 8 | 25 800 | 503 914 | 4 336 | 25 521 | 38 384 | 130 518 | 21 185 | 719 801 | 5 303 | 603 | 844 294 |  |
| 4 | 9 | 0 | 566 903 | 3 258 | 26 600 | 39 843 | 133 631 | 23 342 | 763 720 | 5 500 | 406 | 1 362 278 |  |
| 5 | 9 | 29 025 | 566 903 | 4 878 | 25 494 | 38 349 | 130 454 | 20 616 | 785 347 | 5 298 | 608 | 945 572 |  |

**e-Table 4 III: *R_0_***=2.5

| **Target school** | **Duration (weeks)** | **Cost of lost learning ($1000)** | **Lost productivity due to school closure ($1000)** | **Energy savings ($1000)** | **Health care costs ($1000)** | **Lost productivity due to fatal cases ($1000)** | **Lost productivity due to sickness ($1000)** | **Cost of Oslo municipality ($1000)** | **Total cost ($1000)** | **QALY loss** | **QALY gain (relative)** | **ICER (compared to no intervention) ($ per QALY** | **ICER (relative)** |
| --- | --- | --- | --- | --- | --- | --- | --- | --- | --- | --- | --- | --- | --- |
| 0 | 0 | 0 | 0 | 0 | 32 961 | 47 961 | 155 079 | 32 961 | 236 000 | 6 921 | 0 |  |  |
| 3 | 1 | 3 225 | 0 | 180 | 32 928 | 47 918 | 155 005 | 32 748 | 238 896 | 6 915 | 6 | 468 933 |  |
| 3 | 2 | 6 450 | 0 | 360 | 32 801 | 47 757 | 154 728 | 32 441 | 241 375 | 6 891 | 29 | 182 345 |  |
| 3 | 3 | 9 675 | 0 | 540 | 32 544 | 47 429 | 154 205 | 32 004 | 243 314 | 6 845 | 76 | 96 796 |  |
| 3 | 4 | 12 900 | 0 | 720 | 32 367 | 47 204 | 153 864 | 31 647 | 245 616 | 6 814 | 107 | 90 085 |  |
| 3 | 5 | 16 125 | 0 | 900 | 32 318 | 47 142 | 153 771 | 31 418 | 248 457 | 6 805 | 115 | 108 048 |  |
| 3 | 6 | 19 350 | 0 | 1 080 | 32 308 | 47 130 | 153 752 | 31 228 | 251 460 | 6 804 | 117 | 132 075 |  |
| 3 | 7 | 22 575 | 0 | 1 260 | 32 306 | 47 127 | 153 749 | 31 046 | 254 497 | 6 803 | 117 | 157 565 |  |
| 3 | 8 | 25 800 | 0 | 1 440 | 32 306 | 47 127 | 153 748 | 30 866 | 257 541 | 6 803 | 117 | 183 400 |  |
| 3 | 9 | 29 025 | 0 | 1 620 | 32 306 | 47 127 | 153 748 | 30 686 | 260 586 | 6 803 | 117 | 209 309 |  |
| 2 | 1 | 0 | 26 795 | 188 | 32 929 | 47 921 | 155 022 | 32 741 | 262 479 | 6 915 | 6 | 4 561 284 |  |
| 1 | 1 | 0 | 36 194 | 174 | 32 929 | 47 922 | 155 031 | 32 755 | 271 901 | 6 915 | 6 | 6 390 082 |  |
| 2 | 2 | 0 | 53 591 | 376 | 32 811 | 47 775 | 154 823 | 32 435 | 288 623 | 6 894 | 27 | 1 962 833 |  |
| 4 | 1 | 0 | 62 989 | 362 | 32 899 | 47 884 | 154 977 | 32 537 | 298 387 | 6 910 | 11 | 5 630 857 |  |
| 5 | 1 | 3 225 | 62 989 | 542 | 32 871 | 47 848 | 154 908 | 32 329 | 301 300 | 6 904 | 16 | 3 986 638 |  |
| 1 | 2 | 0 | 72 388 | 348 | 32 797 | 47 760 | 154 839 | 32 449 | 307 436 | 6 892 | 29 | 2 494 487 |  |
| 2 | 3 | 0 | 80 386 | 564 | 32 504 | 47 393 | 154 337 | 31 940 | 314 056 | 6 840 | 80 | 970 117 |  |
| 2 | 4 | 0 | 107 181 | 752 | 32 174 | 46 984 | 153 856 | 31 422 | 339 443 | 6 784 | 137 | 756 410 |  |
| 1 | 3 | 0 | 108 582 | 522 | 32 532 | 47 433 | 154 456 | 32 010 | 342 480 | 6 846 | 75 | 1 422 309 |  |
| 4 | 2 | 0 | 125 979 | 724 | 32 671 | 47 602 | 154 614 | 31 947 | 360 141 | 6 869 | 51 | 2 411 036 |  |
| 2 | 5 | 0 | 133 976 | 940 | 32 013 | 46 784 | 153 631 | 31 073 | 365 463 | 6 757 | 164 | 789 855 |  |
| 5 | 2 | 6 450 | 125 979 | 1 084 | 32 558 | 47 457 | 154 337 | 31 474 | 365 697 | 6 848 | 73 | 1 786 965 |  |
| 1 | 4 | 0 | 144 776 | 696 | 32 315 | 47 166 | 154 148 | 31 619 | 377 709 | 6 808 | 112 | 1 261 624 |  |
| 2 | 6 | 0 | 160 772 | 1 128 | 31 970 | 46 730 | 153 571 | 30 842 | 391 915 | 6 750 | 171 | 911 108 |  |
| 1 | 5 | 0 | 180 970 | 870 | 32 242 | 47 077 | 154 046 | 31 372 | 413 466 | 6 796 | 125 | 1 422 036 |  |
| 2 | 7 | 0 | 187 567 | 1 316 | 31 962 | 46 720 | 153 560 | 30 646 | 418 492 | 6 748 | 173 | 1 057 900 |  |
| 4 | 3 | 0 | 188 968 | 1 086 | 32 117 | 46 916 | 153 760 | 31 031 | 420 675 | 6 773 | 148 | 1 246 918 |  |
| 5 | 3 | 9 675 | 188 968 | 1 626 | 31 806 | 46 514 | 153 021 | 30 180 | 428 358 | 6 715 | 206 | 935 950 |  |
| 2 | 8 | 0 | 214 362 | 1 504 | 31 960 | 46 718 | 153 558 | 30 456 | 445 094 | 6 748 | 173 | 1 210 316 |  |
| 1 | 6 | 0 | 217 164 | 1 044 | 32 226 | 47 057 | 154 023 | 31 182 | 449 427 | 6 793 | 128 | 1 673 032 |  |
| 2 | 9 | 0 | 241 157 | 1 692 | 31 960 | 46 718 | 153 557 | 30 268 | 471 700 | 6 748 | 173 | 1 363 852 |  |
| 4 | 4 | 0 | 251 957 | 1 448 | 31 531 | 46 190 | 152 891 | 30 083 | 481 121 | 6 672 | 249 | 985 746 |  |
| 1 | 7 | 0 | 253 358 | 1 218 | 32 224 | 47 054 | 154 019 | 31 006 | 485 437 | 6 793 | 128 | 1 947 976 |  |
| 5 | 4 | 12 900 | 251 957 | 2 168 | 30 971 | 45 466 | 151 625 | 28 803 | 490 751 | 6 570 | 350 | 726 953 |  |
| 1 | 8 | 0 | 289 552 | 1 392 | 32 223 | 47 053 | 154 019 | 30 831 | 521 455 | 6 793 | 128 | 2 227 690 |  |
| 4 | 5 | 0 | 314 946 | 1 810 | 31 242 | 45 830 | 152 470 | 29 432 | 542 678 | 6 623 | 298 | 1 029 508 |  |
| 5 | 5 | 16 125 | 314 946 | 2 710 | 30 530 | 44 912 | 150 906 | 27 820 | 554 709 | 6 495 | 426 | 747 818 |  |
| 1 | 9 | 0 | 325 746 | 1 566 | 32 223 | 47 053 | 154 019 | 30 657 | 557 475 | 6 793 | 128 | 2 508 535 |  |
| 4 | 6 | 0 | 377 936 | 2 172 | 31 161 | 45 729 | 152 353 | 28 989 | 605 006 | 6 609 | 312 | 1 184 213 |  |
| 5 | 6 | 19 350 | 377 936 | 3 252 | 30 395 | 44 742 | 150 689 | 27 143 | 619 860 | 6 472 | 449 | 854 476 |  |
| 4 | 7 | 0 | 440 925 | 2 534 | 31 145 | 45 709 | 152 330 | 28 611 | 667 575 | 6 606 | 314 | 1 373 129 |  |
| 5 | 7 | 22 575 | 440 925 | 3 794 | 30 360 | 44 699 | 150 633 | 26 566 | 685 399 | 6 466 | 455 | 987 393 |  |
| 4 | 8 | 0 | 503 914 | 2 896 | 31 142 | 45 705 | 152 326 | 28 246 | 730 191 | 6 606 | 315 | 1 569 820 |  |
| 5 | 8 | 25 800 | 503 914 | 4 336 | 30 354 | 44 691 | 150 623 | 26 018 | 751 046 | 6 465 | 456 | 1 128 949 |  |
| 4 | 9 | 0 | 566 903 | 3 258 | 31 142 | 45 705 | 152 325 | 27 884 | 792 816 | 6 606 | 315 | 1 768 143 |  |
| 5 | 9 | 29 025 | 566 903 | 4 878 | 30 353 | 44 689 | 150 621 | 25 475 | 816 713 | 6 464 | 456 | 1 272 225 |  |

e-Table 5 (Scenario F): Cost and health outcome according to type and duration of school closure (The most cost-effective option is highlighted)

| **Target school** | **Duration (weeks)** | **Cost of lost learning ($1000)** | **Lost productivity due to school closure ($1000)** | **Energy savings ($1000)** | **Health care costs ($1000)** | **Lost productivity due to fatal cases ($1000)** | **Lost productivity due to sickness ($1000)** | **Cost of Oslo municipality ($1000)** | **Total cost ($1000)** | **QALY loss** | **QALY gain (relative)** | **ICER (compared to no intervention) ($ per QALY** | **ICER (relative)** |
| --- | --- | --- | --- | --- | --- | --- | --- | --- | --- | --- | --- | --- | --- |
| 0 | 0 | 0 | 0 | 0 | 10 808 | 461 | 59 362 | 10 808 | 70 631 | 1 265 | 0 |  |  |
| 3 | 1 | 3 225 | 0 | 180 | 10 667 | 456 | 58 702 | 10 487 | 72 869 | 1 248 | 16 | 136 427 |  |
| 3 | 2 | 6 450 | 0 | 360 | 10 535 | 450 | 58 091 | 10 175 | 75 166 | 1 233 | 32 | 143 256 |  |
| 3 | 3 | 9 675 | 0 | 540 | 10 391 | 444 | 57 421 | 9 851 | 77 391 | 1 216 | 48 | 139 906 |  |
| 3 | 4 | 12 900 | 0 | 720 | 10 243 | 438 | 56 728 | 9 523 | 79 590 | 1 199 | 65 | 136 789 |  |
| 3 | 5 | 16 125 | 0 | 900 | 10 098 | 432 | 56 049 | 9 198 | 81 804 | 1 182 | 82 | 135 796 |  |
| 3 | 6 | 19 350 | 0 | 1 080 | 9 961 | 427 | 55 401 | 8 881 | 84 059 | 1 167 | 98 | 136 732 |  |
| 3 | 7 | 22 575 | 0 | 1 260 | 9 846 | 422 | 54 857 | 8 586 | 86 439 | 1 153 | 112 | 141 702 |  |
| 3 | 8 | 25 800 | 0 | 1 440 | 9 751 | 418 | 54 406 | 8 311 | 88 935 | 1 142 | 123 | 149 335 |  |
| 3 | 9 | 29 025 | 0 | 1 620 | 9 677 | 415 | 54 056 | 8 057 | 91 553 | 1 134 | 131 | 159 580 |  |
| 2 | 1 | 0 | 26 795 | 188 | 10 602 | 453 | 58 455 | 10 414 | 96 117 | 1 241 | 24 | 1 068 888 |  |
| 1 | 1 | 0 | 36 194 | 174 | 10 712 | 457 | 58 940 | 10 538 | 106 130 | 1 254 | 11 | 3 190 040 |  |
| 2 | 2 | 0 | 53 591 | 376 | 10 444 | 447 | 57 759 | 10 068 | 121 864 | 1 223 | 42 | 1 215 717 |  |
| 4 | 1 | 0 | 62 989 | 362 | 10 508 | 449 | 58 034 | 10 146 | 131 618 | 1 230 | 35 | 1 750 564 |  |
| 5 | 1 | 3 225 | 62 989 | 542 | 10 365 | 443 | 57 359 | 9 823 | 133 839 | 1 213 | 51 | 1 228 395 |  |
| 1 | 2 | 0 | 72 388 | 348 | 10 624 | 454 | 58 555 | 10 276 | 141 674 | 1 243 | 21 | 3 331 376 |  |
| 2 | 3 | 0 | 80 386 | 564 | 10 270 | 440 | 56 989 | 9 706 | 147 521 | 1 202 | 62 | 1 234 563 |  |
| 2 | 4 | 0 | 107 181 | 752 | 10 085 | 432 | 56 167 | 9 333 | 173 114 | 1 181 | 84 | 1 224 582 |  |
| 1 | 3 | 0 | 108 582 | 522 | 10 528 | 450 | 58 132 | 10 006 | 177 170 | 1 232 | 33 | 3 275 314 |  |
| 4 | 2 | 0 | 125 979 | 724 | 10 266 | 440 | 56 962 | 9 542 | 192 921 | 1 202 | 63 | 1 944 398 |  |
| 5 | 2 | 6 450 | 125 979 | 1 084 | 10 005 | 429 | 55 723 | 8 921 | 197 501 | 1 172 | 93 | 1 362 251 |  |
| 2 | 5 | 0 | 133 976 | 940 | 9 897 | 425 | 55 324 | 8 957 | 198 681 | 1 159 | 106 | 1 213 582 |  |
| 1 | 4 | 0 | 144 776 | 696 | 10 427 | 446 | 57 687 | 9 731 | 212 640 | 1 220 | 44 | 3 210 308 |  |
| 2 | 6 | 0 | 160 772 | 1 128 | 9 726 | 418 | 54 554 | 8 598 | 224 341 | 1 139 | 125 | 1 226 562 |  |
| 1 | 5 | 0 | 180 970 | 870 | 10 326 | 442 | 57 243 | 9 456 | 248 112 | 1 209 | 56 | 3 175 639 |  |
| 2 | 7 | 0 | 187 567 | 1 316 | 9 563 | 411 | 53 820 | 8 247 | 250 045 | 1 121 | 144 | 1 245 011 |  |
| 4 | 3 | 0 | 188 968 | 1 086 | 10 009 | 429 | 55 813 | 8 923 | 254 133 | 1 172 | 93 | 1 979 306 |  |
| 5 | 3 | 9 675 | 188 968 | 1 626 | 9 622 | 413 | 53 952 | 7 996 | 261 004 | 1 127 | 138 | 1 382 372 |  |
| 2 | 8 | 0 | 214 362 | 1 504 | 9 434 | 406 | 53 231 | 7 930 | 275 929 | 1 106 | 159 | 1 290 473 |  |
| 1 | 6 | 0 | 217 164 | 1 044 | 10 233 | 438 | 56 829 | 9 189 | 283 621 | 1 198 | 67 | 3 192 509 |  |
| 2 | 9 | 0 | 241 157 | 1 692 | 9 328 | 402 | 52 746 | 7 636 | 301 940 | 1 093 | 171 | 1 349 487 |  |
| 4 | 4 | 0 | 251 957 | 1 448 | 9 739 | 418 | 54 597 | 8 291 | 315 264 | 1 141 | 124 | 1 973 018 |  |
| 1 | 7 | 0 | 253 358 | 1 218 | 10 156 | 435 | 56 485 | 8 938 | 319 215 | 1 189 | 76 | 3 283 500 |  |
| 5 | 4 | 12 900 | 251 957 | 2 168 | 9 226 | 397 | 52 101 | 7 058 | 324 413 | 1 081 | 184 | 1 381 199 |  |
| 1 | 8 | 0 | 289 552 | 1 392 | 10 090 | 433 | 56 194 | 8 698 | 354 877 | 1 181 | 83 | 3 414 059 |  |
| 4 | 5 | 0 | 314 946 | 1 810 | 9 466 | 407 | 53 354 | 7 656 | 376 364 | 1 109 | 156 | 1 964 057 |  |
| 5 | 5 | 16 125 | 314 946 | 2 710 | 8 815 | 380 | 50 154 | 6 105 | 387 711 | 1 033 | 232 | 1 369 569 |  |
| 1 | 9 | 0 | 325 746 | 1 566 | 10 045 | 431 | 55 992 | 8 479 | 390 648 | 1 176 | 89 | 3 615 744 |  |
| 4 | 6 | 0 | 377 936 | 2 172 | 9 210 | 397 | 52 177 | 7 038 | 437 548 | 1 079 | 185 | 1 979 394 |  |
| 5 | 6 | 19 350 | 377 936 | 3 252 | 8 446 | 365 | 48 383 | 5 194 | 451 227 | 990 | 274 | 1 386 886 |  |
| 4 | 7 | 0 | 440 925 | 2 534 | 8 982 | 388 | 51 122 | 6 448 | 498 883 | 1 053 | 212 | 2 022 190 |  |
| 5 | 7 | 22 575 | 440 925 | 3 794 | 8 110 | 351 | 46 755 | 4 316 | 514 922 | 951 | 313 | 1 417 764 |  |
| 4 | 8 | 0 | 503 914 | 2 896 | 8 793 | 380 | 50 241 | 5 897 | 560 432 | 1 031 | 234 | 2 096 006 |  |
| 5 | 8 | 25 800 | 503 914 | 4 336 | 7 811 | 339 | 45 285 | 3 475 | 578 813 | 917 | 348 | 1 459 596 |  |
| 4 | 9 | 0 | 566 903 | 3 258 | 8 639 | 374 | 49 517 | 5 381 | 622 174 | 1 013 | 252 | 2 192 323 |  |
